# Uncovering the Tumorigenic Blueprint of PFOS and PFOA Through Multi-Organ Transcriptomic Analysis of Biomarkers, Mechanisms, and Therapeutic Targets

**DOI:** 10.3390/cimb47090763

**Published:** 2025-09-15

**Authors:** Krisha Mathur, Aleezah Khaliq, Stephanie Park, Nathan Chu, Vaishnavi M. Burra, Norah Kanukolanu, Ellen Costello, Sivanesan Dakshanamurthy

**Affiliations:** 1 The School of Humanities, Arts, and Social Sciences, Rensselaer Polytechnic Institute, Troy, NY 12180, USA; 2 College of Computer, Mathematical, and Natural Sciences, University of Maryland, College Park, MD 20742, USA; 3 College of Arts and Sciences, Georgetown University, Washington, DC 20057, USA; 4 Department of Microbiology, Immunology, and Molecular Genetics, University of California, Los Angeles, CA 90095, USA; 5 Department of Biology, School of Arts & Sciences, College of William and Mary, Williamsburg, VA 23185, USA; 6 Department of Biology, Boston College, Chestnut Hill, MA 02467, USA; 7 Department of Biological Sciences, College of Science, University of Notre Dame, Notre Dame, IN 46556, USA; 8 Department of Oncology, Lombardi Comprehensive Cancer Center, Georgetown University School of Medicine, Washington, DC 20007, USA

**Keywords:** per- and polyfluoroalkyl substances, PFOS, PFOA, mechanisms, tumorigenesis, carcinogenesis, tumor biomarkers, cancer preventive therapeutics, transcriptomics

## Abstract

Per- and polyfluoroalkyl substances (PFASs), called forever chemicals, persist in the environment and bioaccumulate, posing significant health risks. While epidemiological studies have linked exposure to specific PFAS types, perfluorooctanoic acid (PFOA) and perfluorooctane sulfonic acid (PFOS), to an increased incidence of various cancers, specific tumorigenesis mechanisms are unknown. Here, we investigated the potential molecular markers and signatures of perfluorooctane sulfonic acid (PFOS) and perfluorooctanoic acid (PFOA) tumorigenesis. We performed a comprehensive transcriptomic analysis across multiple species and tissue types (*N* = 529) using PFOS and PFOA-exposed RNA-Seq samples. Conserved signatures demonstrate significant disruptions in seven key carcinogenic characteristics including metabolic reprogramming, epigenetic modifications, immune suppression, oxidative stress, and genomic instability. Tumorigenic markers such as *SERPINE1*, *FN1*, *PLIN2*, *ALDOA*, *TRIB3*, and *TSC22D3* and their associated pathways may act independently or synergistically to promote a pro-tumorigenic environment. Additionally, PPARα, LARP1, ACOX1, MYC, and MYCN were identified as key upstream regulators supporting disruptions in lipid metabolism, oxidative stress, and uncontrolled cell proliferation. In liver samples, low concentrations of PFOS and PFOA were sufficient to exhibit tumorigenic signatures associated with tumorigenesis initiation and development. Inferred mechanisms of ccRCC initiation and development were linked to lipid metabolism dysregulation and immunosuppressive signaling. In prostate and testicular xenograft tumor models, carcinogenic mechanisms for tumor progression and promotion were hypothesized. Receptor-mediated signaling and protein synthesis was disrupted in prostate cancer and epigenetic alterations and ECM remodeling observed in testicular cancer. We also explored potential therapeutic rescue strategies, including chemopreventive agents for early intervention. All our findings provide hypotheses for PFOS/PFOA-induced tumorigenesis; however, experimental studies are required to establish translational relevance. All the R codes developed in this study are publicly available.

## 1. Introduction

Per- and polyfluoroalkyl substances (PFASs) are a group of approximately 15,000 synthetic chemicals. They are used in various consumer products due to their high chemical and thermal resistance. PFASs are characterized by strong carbon–fluoride bonds that confer environmental persistence and resistance to degradation. This stability leads to significant bioaccumulation in organisms [1,2]. Perfluorooctanoic acid (PFOA) and perfluorooctanesulfonic acid (PFOS) are the most prevalent forms of PFASs found in the environment [3]. Their prolonged half-life and persistence in the environment have led to bioaccumulation, which is linked to adverse health effects. PFASs are prevalent in water, soil, and the broader environment, and humans are exposed daily through household products, firefighting foams, rainwear, and carpets, leading to widespread environmental contamination [4]. PFAS exposure is evident in the serum of 98% of Americans, posing a significant public health issue [5]. In 2014, the International Agency for Research on Cancer (IARC) classified PFOA as a carcinogen [6]. Numerous epidemiological studies have observed that PFAS exposure and contamination are associated with increased cancer incidence, particularly in the kidney, testicular, liver, breast, ovarian, prostate, non-Hodgkin lymphoma, and thyroid [7,8,9,10,11]. Empirical studies have found that PFOS and PFOA exposure causes epigenetic alterations, disrupts cellular signaling pathways, impairs immunoregulation, and facilitates carcinogenesis [12,13]. The carcinogenic potential of PFOS and PFOA exposure is correlated with cancer initiation, progression, and metastasis [14].

Evidence from previous studies demonstrates a strong association between PFOS and PFOA exposure and epigenetic changes, such as DNA methylation, histone modifications, and miRNA expression, which are linked to adverse health outcomes [15]. These alterations suggest that PFAS exposure can initiate or sustain pro-carcinogenic states by activating oncogenes and promoting genetic instability [12,16]. Additionally, PFOS and PFOA exposure have been shown to induce chronic inflammation, characterized by the persistent recruitment of pro-inflammatory immune cells that increase the production of reactive oxygen species and oxidative stress [17]. These mechanisms promote instability in the tumor microenvironment and interact with cell signaling pathways implicated in tumorigenesis [18,19]. Other studies have found links between PFAS exposure and the circulation of inflammation and oxidative-stress-associated biomarkers [19,20]. These studies have assessed the mechanisms behind PFOS exposure and the overexpression of biomarkers that increase the tumor microenvironment conductivity to cancer development. Still, an in-depth categorization and assessment of specific biomarkers related to multiple PFAS-type exposures are missing. PFOA and other PFASs modulate cellular signaling mechanisms such as hormonal and lipid signaling. These types of signaling promote cancer development and progression by altering gene transcription [21]. Further evidence supports that PFOS and PFOA induce cellular alterations such as cell migration, invasion, and increased proliferation in varying tissue cancerous cells [21,22,23].

Given the prevalence, persistence, and known carcinogenic potential of PFOS and PFOA, further investigation into tumorigenic mechanisms is needed. While previous studies link PFASs to cancer, their mechanisms driving tumorigenesis remain poorly understood. This study seeks to understand PFOS and PFOA tumorigenic mechanisms of initiation, development, progression, and promotion. We established a cross-species and cross-tissue analysis of samples from mice, humans, and monkeys. We carried out transcriptomic profiling of non-tumor and tumor tissues to identify differentially expressed genes and pathways associated with PFOS and PFOA-induced tumorigenicity. We explored how PFOS and PFOA promote tumorigenesis by disrupting key molecular mechanisms that may independently or collectively establish a pro-tumorigenic environment [15,24]. We also identified therapeutic targets based on conserved biomarkers, tumor-specific targets, and chemopreventive strategies.

## 2. Materials and Methods

### 2.1. Data Acquisition, Processing, and Preparation

**Figure 1:**
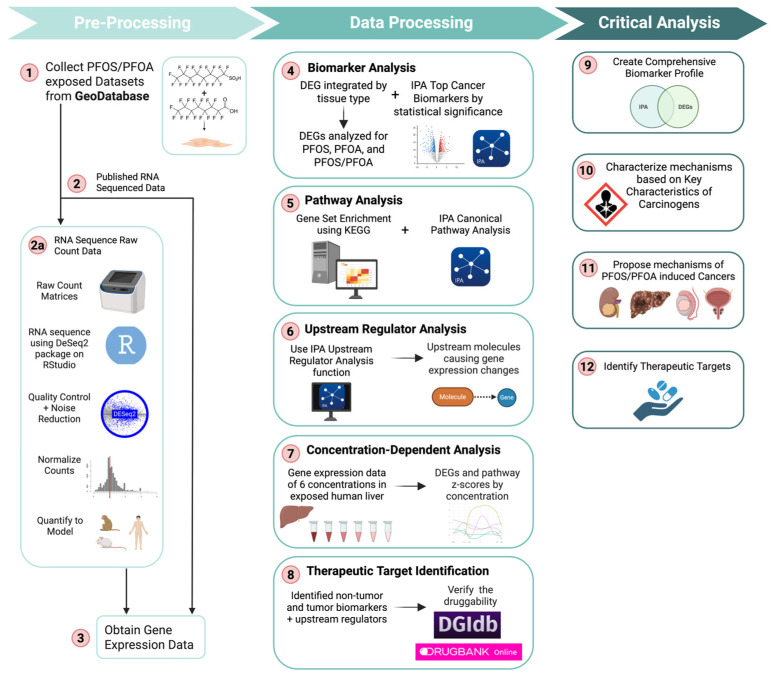
An illustration of the workflow to investigate the tumorigenic effect of PFOS and PFOA exposure on pathways and biomarkers to elucidate carcinogenic mechanisms and propose therapeutic strategies. Cross-tissue and species datasets were acquired through a search on the GEO database for PFOS and PFOA-exposed samples (Step 1). Datasets were then RNA-sequenced using DESeq2 in R (Step 2 and 2a). After sequencing, RNA-seq gene expression data and the published gene expression data were organized into a spreadsheet (Step 3). RNA-seq data went through two methods of biomarker analysis, including a differential gene expression analysis by tissue and PFAS type using RStudio and identification of top cancer biomarkers using Ingenuity Pathway Analysis (IPA) (Step 4). Imported RNA-seq data were processed through IPA’s pathway analysis to determine highly regulated canonical pathways and a gene set enrichment analysis (GSEA) to determine altered pathways using gene expression (Step 5). An upstream regulator analysis was run to identify additional significant molecules causing gene expression changes (Step 6). Gene expression changes across six PFOS and PFOA concentrations were evaluated by plotting the number of DEGs and pathway z-scores at each dose for analysis of concentration specific effects on liver toxicity (Step 7). Significant DEGs were filtered for therapeutic relevance, and druggability was assessed using the Drug Gene Database and DrugBank (Step 8). For critical analysis, the biomarker data were all combined and analyzed to create a comprehensive biomarker profile (Step 9). Biomarkers, pathways, and upstream regulators were characterized using the key characteristics of carcinogens framework and analyzed to determine cancer mechanisms of PFOS and PFOA exposure (Step 10). The tumor and tissue specific data were analyzed to propose cancer specific mechanisms for clear cell renal cell carcinoma (ccRCC), liver, testicular, and prostate cancers (Step 11). Potential gene targets and carcinogenic mechanisms were evaluated for therapeutic applications (Step 12). Each step is detailed further in the following methods section.

Eight PFOS/PFOA-exposed datasets containing 529 samples from different species and tissue types were obtained from the Gene Expression Omnibus (GEO) database (Figure 1; Table 1). These datasets included samples from Homo sapiens (470), Mus musculus (49), and Macaca mulatta (10). We analyzed tumor and non-tumor tissues exposed to PFOS and PFOA. The non-tumor data were examined to identify pathways and biomarkers indicative of tumor initiation and development. For tumor samples, we included a testicular germ cell tumor dataset and a prostate tumor dataset (Table 1). These tumor samples were analyzed to identify pathways and biomarkers associated with tumor progression and metastasis. The categorization and analysis methods applied to tumor samples mirrored those used for non-tumor samples. These results were specifically evaluated for evidence of tumor advancement and metastatic potential. Statistical analyses were conducted in R statistical software version 4.3.1 and Bioconductor version 3.18. All the R code used for RNA sequencing, molecular signature analysis, and figure generation are available at the following address: https://github.com/sivaGU/PFOS-PFOA-Exposure-Analysis (accessed on 5 August 2025). The following packages were used to perform the analysis and generate figures for the pathway analysis: *ggplot2* R package version 3.5.2, *enrichplot* R package version 1.22.0, and *clusterProfiler* R package version 4.10.1. Biorender.com was used for figure generation in this study.

### 2.2. RNA Sequencing with RStudio to Generate Differentially Expressed Genes

Several datasets (GSE144775, GSE262137, GSE208636, GSE119441, GSE212294) did not publish the RNA-sequenced data. In this case, we downloaded the raw counts for RNA sequencing and processing. The *DESeq2* R package version 1.42.1 was employed to sequence each of the PFOS/PFOA-exposed samples. We obtained the log_2_ fold change, false discovery rate (FDR), and *p*-values. Low-expressing genes (less than 10 total counts across samples) were filtered out to reduce noise. The DESeq2 workflow was applied to normalize counts and identify differentially expressed genes (DEGs). Gene annotation and identifier mapping was performed using the *AnnotationDbi* R package version 1.64.1 along with the organism specific *org.Hs.eg.db* R package version 3.18.0 and *org.Mm.eg.db* R package version 3.18.0 annotation databases. For GSE144775, multiple concentrations of PFOS and PFOA exposure were tested (0.02–100 μM), and the highest concentrations (50 and 100 μM) were cytotoxic to cells. Therefore, we decided to retain only the 20 μM of PFOS and PFOA concentration in our comparison analysis, as it was reported to be the top non-cytotoxic concentration across all PFAS concentrations analyzed with the highest number of DEGs [26]. To analyze concentration-dependent responses, we sequenced and analyzed all non-cytotoxic concentrations (0.02, 0.1, 0.2, 1, 10, 20 μM) to make conclusions about toxicity and carcinogenesis in the liver. For GSE212294, the Mouse Ensembl gene IDs were converted to gene symbols [32]. This dataset included samples treated with 0.05 and 0.3 mg/kg/day of PFOA in PPARα knockout and wildtype mice. Although all the data were sequenced, only the wildtype mice treated with PFOA were used to make carcinogenic conclusions in this study [30]. To compare datasets, we compiled fold change values for each RNA-sequenced sample under a single gene list and filtered for differentially expressed genes with a *p*-value < 0.05 (Excel Table S1).

#### 2.2.1. Validation of RStudio RNA-Sequencing Workflow

The RNA-seq workflow was validated using the GSE236956 dataset by comparing processed raw counts with the DeSeq2 workflow (yellow column) with published results (green column). The differences between the datasets are all below 0.1 (purple column), confirming the accuracy of this study’s RNA-seq workflow. This was performed to confirm the reliability of workflow used (Table 2).

#### 2.2.2. Batch Effect Mitigation and Meta-Analysis Validation

The datasets in this study differed in species, tissue type, and exposure conditions. To mitigate these batch effects, we analyzed each dataset with available raw counts independently using a standardized DESeq2 workflow. This ensured that the differential expression results were reflected within dataset contrasts and avoided confounding effects that could arise from merging raw counts across various experimental systems [33]. Correlation between individual datasets was analyzed by calculating the pairwise Pearson correlation coefficients using *corrplot* R package version 0.95. In addition, we integrated results via *p*-value meta-analysis using the *metaRNASeq* R package version 1.0.8 package in RStudio to assess the robustness of our findings [33]. A combination of the inverse-normal Stouffer method, which accounts for study sample sizes, and Fisher’s method, which is sensitive to strong effects in individual studies, was performed. Replicate counts were used as study weights and FDR was controlled by the Benjamini–Hochberg procedure. Significant genes from the meta-analysis (filtered by FDR < 0.05) were compared with a set of robust genes from the individual analyses. Overlapping genes were identified between the individual and meta-analysis. Additionally, a pathway enrichment analysis using Hallmark and Reactome gene sets was performed on the meta-analysis genes. The packages *clusterprofiler* R package version 4.10.1 and *msigdbr* R package version 25.1.0 were used.

### 2.3. Tumor Biomarker Discovery

To comprehensively assess the transcriptome-wide response to PFOS and PFOA, we designed a comprehensive profile from two distinct biomarker analyses. For the first analysis, we used QIAGEN Ingenuity Pathway Analysis (IPA) (QIAGEN, Hilden, Germany). The biomarker filter was applied to identify biomarkers in the non-tumor samples based on significance levels of *p*-value < 0.05 and FDR < 0.05. We filtered specifically for cancer-related biomarkers, looking at both known and unclassified types using the IPA knowledgebase. Initial analysis identified 6 to 350 biomarkers per dataset, then we applied conditional formatting in Excel to identify biomarkers present in at least 6 out of 8 datasets, resulting in a final set of 20 biomarkers linked to either PFOS or PFOA exposure. In the second analysis, we used RStudio to identify and investigate differentially expressed genes present in samples within each dataset to determine which genes were consistently up or downregulated in response to PFOS/PFOA exposure across tissue types: human liver, human stem cells, monkey stem cells, and mouse liver. For tissue types with multiple datasets, we applied a weighted Stouffer integration to balance the contributions from each tissue and prioritize key genes that were differentially expressed across datasets [14]. This analysis identifies significantly upregulated and downregulated genes present in at least three of the six tissue types, showing insight into genes potentially involved in PFOS/PFOA-induced carcinogenicity.

#### Tumor Biomarker Categorization

The goal of the biomarker analysis was to develop a comprehensive profile of cancer-associated biomarkers correlated with PFOS and PFOA exposure to determine related cancer types and understand the implications of altered biological functions as a result of PFOS and PFOA exposure. Once the biomarkers were identified, we analyzed and conducted literature reviews of each biomarker to investigate the biological functions and their impact and association with cancer. Significant biomarkers were further analyzed to develop a mechanistic understanding of their role in cancer influenced by PFOS and PFOA exposure. Additionally, cancer types associated with biomarkers were identified and categorized through a literature review of each biomarker to determine the most highly correlated cancer types with PFOS and PFOA exposure for mechanistic comprehension.

### 2.4. Identification of Significant Pathways

We employed IPA to determine the most significant expressed canonical pathways across all PFOS/PFOA-exposed samples. Each RNA-sequenced PFOS/PFOA dataset was imported into IPA. To identify significant pathways associated with the patterns of differential gene expression, core analyses were run in IPA separately for each dataset, with a significant *p*-value < 0.05. The core analysis feature within IPA allowed us to determine and identify significant pathways, upstream regulators, and top-expressed diseases. The core analysis tool generated the top down and upregulated canonical pathways for each dataset based on the DEGs. After the core analysis function was performed, the comparison analysis tool in IPA was used to compare the top upregulated and downregulated pathways across the PFOS and PFOA-exposed non-tumor samples to identify conserved pathways.

In addition to the identification of interaction network pathways, we expanded our pathway analysis through the utilization of a gene set enrichment analysis (GSEA). We performed a GSEA using the Kyoto Encyclopedia of Genes and Genomes (KEGG) pathways database in RStudio. The GSEA was employed on the non-tumor samples to determine the top enriched pathways conserved across samples. Then, the top enriched pathways were determined based on the number of differentially expressed genes within a pathway (fold change > ±2) and then the pathways present in at least two of the datasets were compiled and analyzed based on the Normalized Enrichment Score (NES).

### 2.5. Identification of Significant Upstream Regulators and Downstream Effects

To identify upstream molecular regulators and targets that drive gene expression changes, we used the upstream regulator analysis function in IPA [34]. The top regulators and their expression levels were cross referenced with Comparative Toxicogenomics Database (CTD) [35]. Each regulator included in the final analysis demonstrated similar expression levels in CTD and were supported by at least two or more references. Additionally, we used the downstream effects function in IPA to examine predicted downstream biological functions, toxicological functions, and diseases. Diseases and functions with a z-score greater than 0 are referred to as “enriched” and those with z-scores lower than 0 were considered “diminished”.

### 2.6. Identification of Therapeutic Targets as a Rescue Strategy

To identify novel therapeutic targets that can rescue the potential hazardous effects of PFOS and PFOA exposure, conserved DEGs from the biomarker analysis were filtered by the greatest overall fold change across the non-tumor datasets. Significant upstream regulators were cross referenced with the literature to evaluate their potential as therapeutic targets for tumor prevention. For tumor-specific treatments, biomarkers from prostate and testicular tumor samples were similarly ranked by fold change to identify tumor-specific targets. Mechanistic targets based on significant pathways and biomarkers associated with tumor-specific mechanisms were additionally investigated. The Drug–Gene Database and DrugBank version 5.1.13 was then used to verify and validate conclusions made using information about druggability for the potential targets [36,37]. These genes were then combined into a comprehensive list of therapeutic targets, along with the identified therapeutic biomarkers.

## 3. Results and Discussion

### 3.1. Correlation Analysis and Batch Effect Assessment

The correlation matrix analysis demonstrated that PFOS and PFOA exposures within the same species and tissue type exhibited strong positive correlations (Figure S1). Similarly, interspecies comparisons of the same tissue type revealed notable positive correlations. This indicates that PFOS and PFOA-induced transcriptomic responses are conserved across species and not necessarily driven by dataset-specific batch effects. In addition, the *p*-value combination meta-analysis performed by species yielded results consistent with the independent DESeq2 analyses, further supporting the reliability of the conserved transcriptomic responses discussed in the following sections [33]. The results of the meta-analysis are further outlined in Supplementary Section S1.

### 3.2. Identification of PFOS/PFOA-Associated Cancer Biomarkers

**Figure 2:**
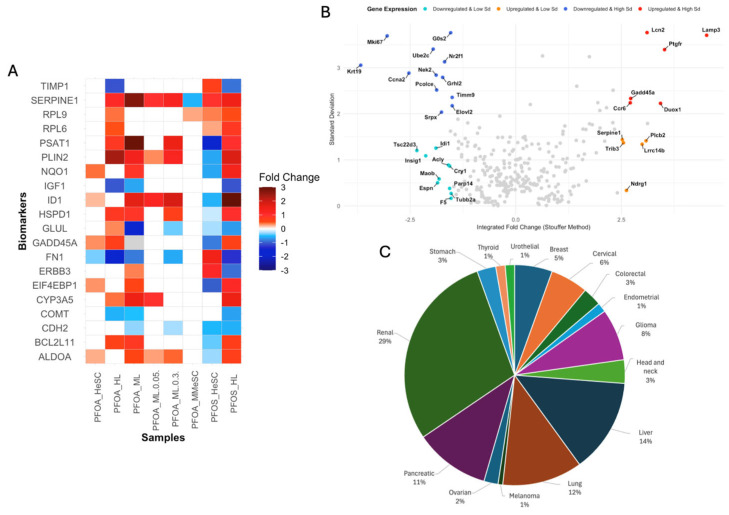
Conserved biomarkers and their cancer association. (**A**) DEGs filtered by FDR, *p*-value, and cancer association (present in >five of eight non-tumor datasets) are presented in the heatmap with the fold change values of each biomarker. The red color indicates upregulation (fold change > 0), blue color indicates downregulation (fold change < 0), and white indicates no calculated fold change. (**B**) Volcano plot of gene expression values across PFOS/PFOA samples, present in at least 3/4 tissue types. The colors of the top up and downregulated genes are specified by the legend on the top. Standard deviation (SD) values are shown on the y-axis, with significant SD values (>2) differentiated by color or the point. (**C**) Frequency of cancer subtypes associated with the 53 biomarkers identified (*p*-value < 0.05) from the PFOS/PFOA-exposed non-tumor samples, with renal, liver, pancreatic, and lung cancer showing highest associations.

For biomarker profiling, two approaches were used: IPA’s biomarker filter and weighted Stouffer integration of tissue-specific DEGs. First, twenty genes were identified with differential expression across non-tumor samples using stringent statistical filters (Figure 2A). Secondly, 33 genes were significantly differentially expressed across different tissue types, with 11 upregulated and 22 downregulated (Figure 2B). Separate analyses were conducted for non-tumor samples exposed to PFOS, PFOA, or both compounds. Specific DEGs for PFOA exposure included *COL16A1*, *PDGFR*, *DDR2*, and *TRBC1*, while PFOS-specific DEGs included *CIDEC*, *HSD17B11*, *PEX11A*, and *PFI1A* (Figure S2) (Table S1). The 53 biomarkers were categorized by cancer association and have potential to serve as prognostic markers for renal, liver, pancreatic, lung, glioma, breast, colorectal, cervical, endometrial, and stomach cancers (Figure 2C) [38].

The biomarkers with strong evidence of cancer association were compiled to create a comprehensive biomarker profile with 30 genes (Table S1). A majority of these genes were involved with metabolic reprogramming, immune modulation, and extracellular matrix (ECM) remodeling [39,40,41]. Highly notable biomarkers, *FN1* and *SERPINE1*, were identified as DEGs in six of eight datasets. FN1 was downregulated across samples, being involved in ECM integrity (Figure S4). This suggests a weakening of structural tissue barriers [42,43,44,45,46]. *SERPINE1* was upregulated; as a key regulator of angiogenesis and apoptosis, it contributes to creating a tumor supportive microenvironment (Figure S3) [47,48,49,50,51,52,53,54]. *ALDOA*, *TRIB3*, *PLIN2*, *ID1*, and *TSC22D3* genes also exhibited differential expression patterns indicative of carcinogenic mechanisms. *ALDOA* and *PLIN2* were both upregulated and have been previously associated with enhanced glycolysis and lipid accumulation, both hallmarks of cancer metabolism (Figures S6 and S7) [55,56,57,58]. *TRIB3* is involved with stress signaling and survival under hypoxic conditions (Figure S8) [59,60]. *ID1* (upregulated) and *TSC22D3* (downregulated) are both involved in immune regulation, suggesting immunosuppressive mechanisms that contribute to immune evasion and tumor progression (Figures S5 and S10) [61,62,63,64,65]. These results suggest that exposure to PFOS and PFOA modulates gene expression involved in multiple cancer-associated processes (Supplementary Section S2). To further study the functional roles of these biomarkers, we categorized them based on their predicted role in tumorigenicity. These are discussed in the following sections.

### 3.3. PFOS/PFOA Molecular Markers and Signatures Defined by the Key Characteristics of Carcinogens Framework

The results of biomarker profiling (Excel Table S2) and pathway analysis (Excel Table S3) in non-tumor samples revealed molecular signatures reflective of tumor initiation and development. These findings were evaluated within the framework of the IARC key characteristics of carcinogens [16]. The key mechanisms identified include induction of epigenetic alterations, oxidative stress, and chronic inflammation; immunosuppression; modulation of receptor-mediated effects; and alterations in cell proliferation, cell death, and nutrient supply (Table 3). These key characteristics are further discussed in the following sections.

#### 3.3.1. PFOS/PFOA Exposure Modulates Receptor-Mediated Effects

**Figure 3:**
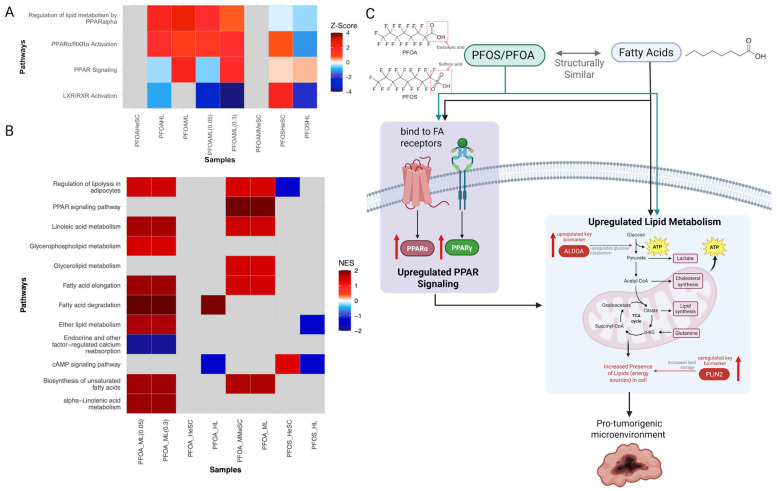
Pathways related to PFOS/PFOA modulating receptor-mediated effects and their potential mechanisms of carcinogenicity. (**A**) Heatmap of top receptor-mediated effects pathways from Regulatory Network Pathway Analysis. (**B**) Heatmap of top receptor-mediated effects pathways from GSEA. (**C**) Visual representation of the key carcinogenic dysregulated pathways and genes in response to the disruption of fatty acid signaling and lipid metabolism pathways by the structural similarity of PFOS/PFOA to fatty acids.

PFOS and PFOA exposure upregulated PPAR receptor signaling across all samples, with PFOA additionally disrupting fatty acid metabolism pathways (Figure 3A,B). Pathways of fatty acid degradation, elongation, biosynthesis, short chain fatty acid metabolism, and specific fatty acid metabolism were all upregulated in PFOA samples (Figure 3B). Due to the structural similarity of PFASs to fatty acids, they may bind to and act as ligands for the same nuclear receptors (Figure 3C) [15]. This may explain the observed modulation of PPAR signaling and LXR/RXR activation pathways (Figure 3A,B) [66]. Benninghoff et al. and Roy et al. found that PFOS and PFOA can modulate PPAR signaling by binding to either the normal ligand binding pocket or to allosteric binding sites on the receptor [67,68]. Moreover, Vanden Heuvel et al. showed PFOS and PFOA target PPARα and PPARγ, but neither compound directly activates LXRs [69]. Therefore, the observed change in LXR and PPAR function can also be a result of PFOS/PFOA-induced metabolism of naturally occurring fatty acid ligands.

Further, biomarkers such as *LDLR* and *ALDH3A2* were found to be upregulated (Table S1; Figure S9). The upregulation of these genes indicates an increase in lipoprotein receptor activity and fatty acid oxidation. These changes suggest that PFOS and PFOA may modulate receptor and enzyme activation associated with lipid metabolism. *PLIN2*, another upregulated key biomarker, is involved in the synthesis and storage of lipid droplets within cells (Figure S7). The upregulation of *PLIN2* contributes to lipid accumulation as the rate of formation and the amount of lipid droplets increases [70]. A buildup of excess lipids within the liver causes hepatocellular diseases that are risk factors for cancer development, as they exhibit a metabolic environment suitable for tumor growth (Figure 3C).

#### 3.3.2. PFOS and PFOA-Induced Alterations in Cell Proliferation, Cell Death, and Nutrient Supply

**Figure 4:**
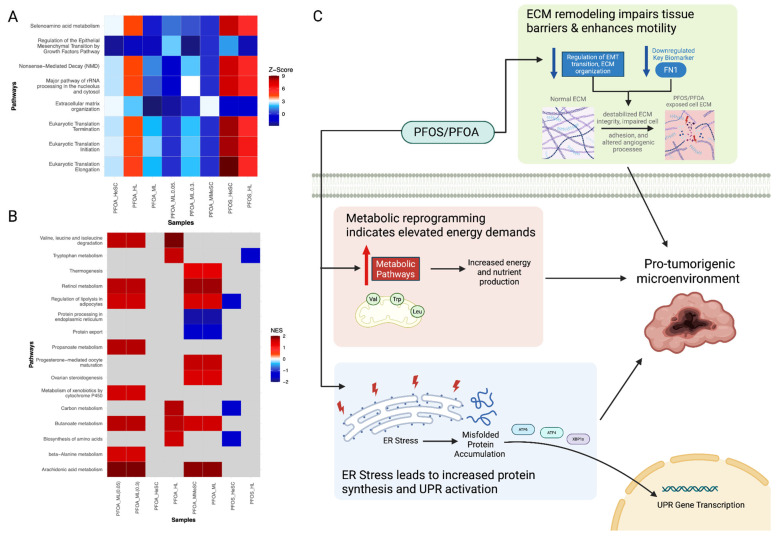
Pathways of PFOS/PFOA-altered cell proliferation, cell death, and nutrient supply and their potential mechanisms of carcinogenicity. (**A**) Heatmap of top pathways from Regulatory Network Pathway Analysis. (**B**) Heatmap of top pathways from GSEA. (**C**) Visual representation of PFOS/PFOA inducing ER stress, ECM remodeling, and metabolic reprogramming, which collectively impair protein folding, destabilize tissue structure, and alter cellular metabolism, fostering conditions for tumor initiation and progression.

Branched-chain amino acid (valine, leucine, and isoleucine) degradation pathways were upregulated by PFOS and PFOA exposure (Figure 4A,B). Sivanand et al. demonstrated upregulated branched-chain amino acid pathways can disrupt protein synthesis, epigenetic regulation, and mitochondrial biogenesis, contributing to carcinogenicity (Figure 4C) [71]. Additionally, amino acid metabolism pathways were consistently upregulated across PFOS and PFOA-exposed samples (Figure 4B). The expression of the key biomarkers *GLUL* and *PSAT1* were also disrupted, both of which are involved in amino acid metabolism (Table S1) [72,73]. Lieu et al. found that these alterations in amino acid metabolism occur in cancer to maintain energy production and redox balance [74]. Further, protein translation pathways, including eukaryotic translation initiation, elongation, and termination, were consistently upregulated in exposed samples (Figure 4A). This is a result of the overexpression of EIF genes, which indicate increased protein production [75] (Figure 4A). Ribosomal proteins, such as *RPL9* and *RPS6*, were similarly upregulated. Both have been shown to be overexpressed in various cancer cells and are associated with development and progression of malignant cancers (Table S1) [76]. Similarly, Hwang et al. reported alterations in ribosomal biogenesis can promote tumorigenesis by increasing translational capacity [77]. However, this increased translational burden can overwhelm the ER protein-folding machinery, as indicated by the downregulation of ER protein processing and export pathways (Figure 4B,C) [78]. Shaheen et al. reported that PFOS and PFOA exacerbate ER stress and activate the unfolded protein response (UPR), a pathway associated with tumor progression (Figure 4C) [79,80].

The downregulation of key ECM-related biomarkers, including *SDC1* (cell adhesion and signaling), *ACTA1* (cytoskeletal structure), and *CDH2* (cell–cell adhesion) reflects impaired ECM remodeling and weakened tissue structure (Table S1). Notably, *FN1*, a critical ECM glycoprotein, was consistently downregulated across exposed samples (Figure S4). The destabilization of ECM structure and impairment of cell adhesion caused by *FN1* suppression promotes a permissive tumor microenvironment [81]. Such changes in ECM dynamics can increase cell motility and angiogenesis, which may promote tumor invasion and progression, consistent with Prakash et al. [82]. Taken together, PFOS and PFOA exposure can contribute to a pro-tumorigenic microenvironment through protein synthesis and ECM structure alterations, which alter cell proliferation, cell death, and nutrient supply (Figure 4C).

#### 3.3.3. PFOS and PFOA-Induced the Suppression of Immune and Inflammatory Responses

**Figure 5:**
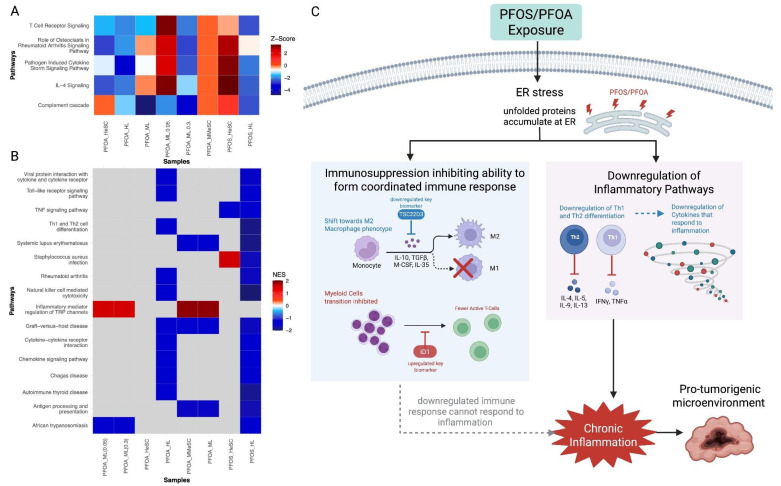
Pathways related to PFOA/PFOA inflammatory and immune response and their potential mechanisms of carcinogenicity. (**A**) Heatmap of top inflammatory and immune response pathways from Regulatory Network Pathway Analysis. (**B**) Heatmap of top pathways from GSEA. (**C**) Visual representation of the key carcinogenic dysregulated pathways and genes involved in immune system and inflammatory dysfunction when samples were exposed to PFOS and PFOA. The inflammation induced by the downregulation of inflammatory pathways and the inability of PFOS/PFOA-exposed samples to generate a coordinated immune response to the inflammation leads to unresolved chronic inflammation, creating an immunosuppressive, pro-tumorigenic microenvironment.

A majority of the inflammatory and immune response pathways were downregulated in response to PFOS and PFOA exposure (Figure 5A,B). This suggests an impaired immune system and active tumor microenvironment (Figure 5C). Consistently, Zhang et al. demonstrated that the presence of PFOS and PFOA in cells impairs immune function and alters cytokine signaling involved in inflammation (Figure 5C) [17,83]. Additionally, pathways of complement cascade, Th1 and Th2 cell differentiation, and antigen processing and presentation pathways were downregulated (Figure 5A,B) [84]. PFOS and PFOA exposure disrupts the complement cascade pathway, which prevents abnormal cells from being marked for destruction. Thus, PFOS and PFOA-exposed tumor cells can evade detection (Figure 5C). Furthermore, autoimmune disease pathways including graft-versus-host disease, systemic lupus erythematosus, autoimmune thyroid disease, and rheumatoid arthritis are consistently downregulated (Figure 5B). These alterations suggest a loss of immune tolerance potential, which makes tissues and organs more prone to chronic inflammation and tumor development, as shown by Mackay et al. (Figure 5C) [85]. The persistent inflammation, rather than being protective, can result in a microenvironment that allows for tumor development (Figure 5C) [86].

Moreover, *TSC22D3* was significantly downregulated and *ID1* was highly upregulated (Figures S5 and S10). *TSC22D3* modulates macrophage polarization and when downregulated, shifts toward a M2 macrophage phenotype. M2 macrophages have immunosuppressive functions and secrete anti-inflammatory cytokines that allow for tumor invasion as well as hinder immune surveillance [62]. *ID1* inhibits the transition of myeloid cells to antigen presenting cells. Hence, its upregulation leads to an increased amount of myeloid cells that inhibit T cell activation. The combined downregulation of immune and inflammatory responses contribute to chronic tissue damage and the inability to control tumor growth due to the lack of immune surveillance (Figure 5C).

#### 3.3.4. PFOS/PFOA Disrupts Mitochondrial Signaling and Induces Elevated Oxidative Stress

**Figure 6:**
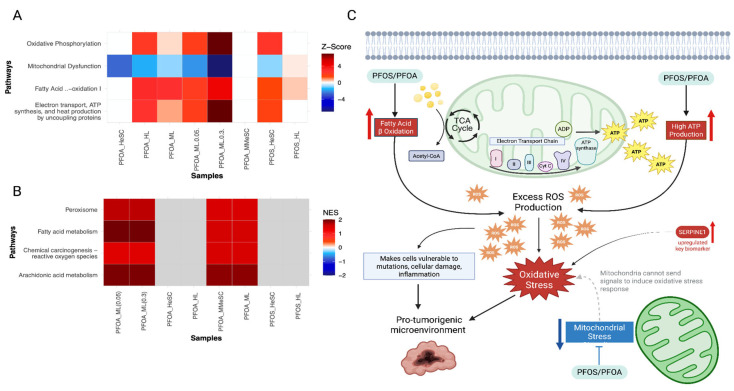
Pathways related to PFOS/PFOA-induced oxidative stress and their potential mechanisms of carcinogenicity. (**A**) Heatmap of top oxidative stress response pathways from Regulatory Network Pathway Analysis. (**B**) Heatmap of top oxidative stress response pathways from GSEA. (**C**) Depiction of affected pathways related to intracellular ROS buildup. Increased levels of ATP production and fatty acid beta oxidation increase ROS, which broadly causes cellular damage. Analysis shows mitochondrial stress signaling is blocked, inhibiting oxidative stress response from decreasing ROS concentrations, creating a pro-tumorigenic microenvironment.

Pathways associated with oxidative phosphorylation, electron transport, and fatty acid beta oxidation were upregulated (Figure 6A,B). This suggests an increased production of reactive oxygen species (ROS) (Figure 6C) [87]. The increased metabolic activity observed can also contribute to the increased production of ROS (Figure 6C) [88]. This accumulation of ROS may induce oxidative damage to the DNA, RNA, proteins, lipids, and mitochondria, which can be carcinogenic [87]. In normal cells, the mitochondria signals an oxidative stress response to reduce ROS levels. However, in the analysis, a decreased level of mitochondrial signaling was observed (Figure 6A). PFOS and PFOA can disrupt mitochondrial function and decrease the mitochondrial ability to detect and respond to the high levels of ROS (Figure 6C). This makes the cell prone to damage, inflammation, DNA alterations, and supports an environment suitable for tumor progression [89].

The downregulated biomarker *TRIB3* can interact with lipid-associated proteins, such as *PLIN2*, and exacerbate ROS production through lipid peroxidation (Figures S6 and S7) [90]. *SERPINE1* also contributes to the accumulation of ROS by increasing hypoxia and inflammation within the tumor microenvironment (Figure S3) [91]. Therefore, the observed changes suggest PFOS and PFOA-induced oxidative stress that promotes cellular damage and supports tumorigenesis (Figure 6C).

#### 3.3.5. PFOS/PFOA-Induced Epigenetic Reprogramming and Genomic Instability Drive Tumor Promotion

**Figure 7:**
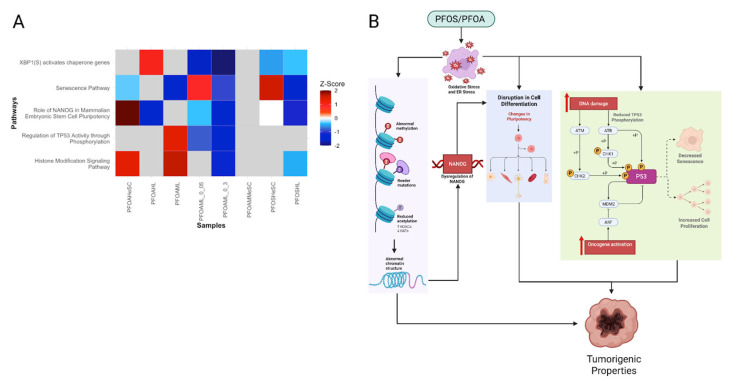
Pathways related to PFOS/PFOA-induced epigenetic reprogramming and genomic instability and their potential mechanisms of carcinogenicity. (**A**) Heatmap of top pathways from Regulatory Network Pathway Analysis. (**B**) Visual representation of histone modifications and genomic instability mechanisms disrupted that promote a pro-tumorigenic environment.

PFOS and PFOA interfere with the epigenetic landscape of cells, which in turn disrupts tumor suppressor and oncogene expression. Alterations in histone modification signaling suggest changes in chromatin structure that contribute to the changes in gene expression of tumor suppressor genes or oncogenes (Figure 7A) [92]. Additionally, the role of *NANOG* in mammalian stem cell pluripotency pathway was dysregulated (Figure 7A). As shown by Hattori et al., epigenetic changes such as DNA methylation and chromatin remodeling can suppress *NANOG* transcription [93]. Silencing of *NANOG* may alter the pluripotency of cells, which in turn disrupts cell differentiation (Figure 7A) [93]. Together, these histone-related changes influence the epigenome and alter the expression of cancer-related genes, which promotes cell proliferation and cancer growth (Figure 7B).

Moreover, PFOS and PFOA downregulated cellular senescence, generally maintained by stable epigenetic functions to enforce permanent cell cycle arrest (Figure 7A) [94]. However, the observed reduced *TP53* phosphorylation activity can impair senescence, which leads to uncontrolled cell proliferation, as demonstrated by Yamamoto et al. (Figure 7A) [95]. Downregulation of *XBP1* activity compromises proteostasis, leading to misfolded protein accumulation and furthering genomic instability (Figure 7A) [96]. We also noted an upregulation of *GADD45A* (Table S1). Increased exposure to PFOS and PFOA leads to chronic activation of this gene, which contributes to alterations in DNA methylation and chromatin remodeling. Collectively, these epigenetic alterations silence the growth inhibitory pathways and reprogram the gene expression. Such alterations allow cancer cells to evade apoptosis and continuously proliferate, in turn driving tumor initiation and progression (Figure 7B).

#### 3.3.6. Alterations in the Upstream Regulators PPARα, LARP1, ACOX1, MYC, and MYCN Contribute to Increased Cell Proliferation

**Figure 8:**
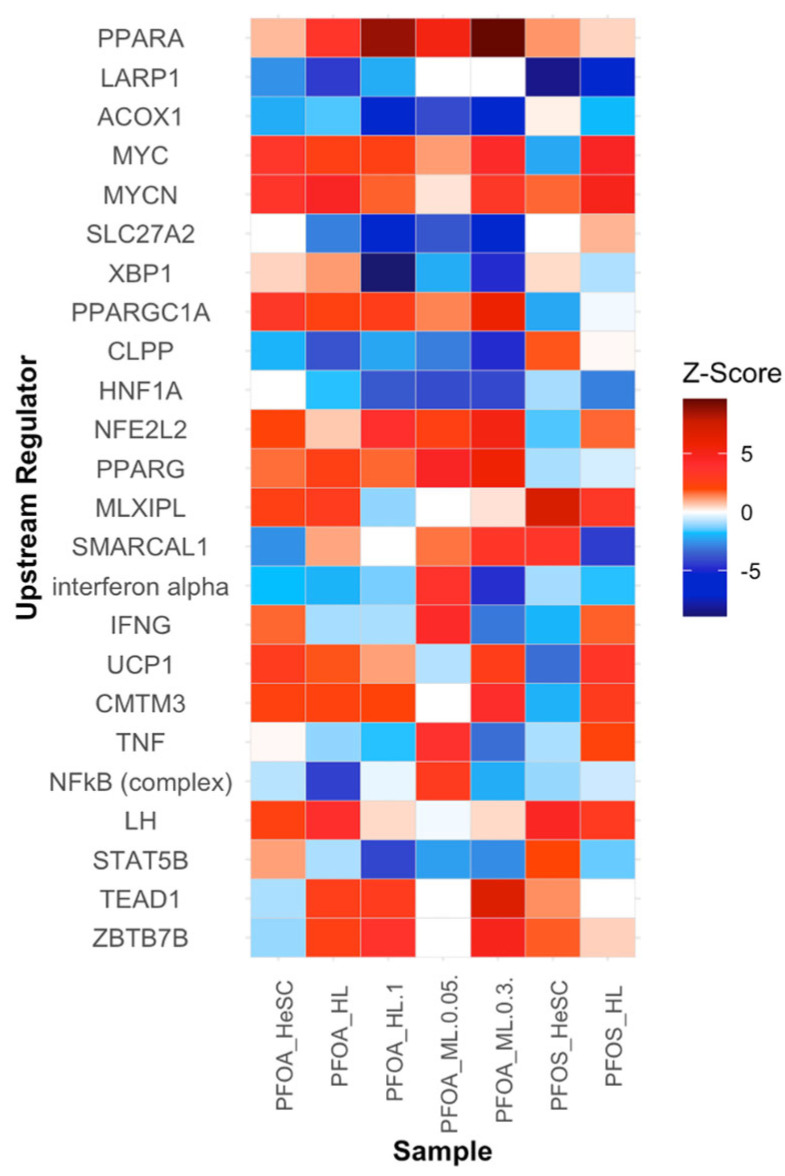
Upstream regulator expression levels across all datasets. PPARa demonstrated the highest expression levels and was upregulated in all datasets minus the PFOA monkey stem cells. Additional significant genes that showed altered expression patterns were LARP1, ACOX1, MYC, MYCN, SLC27A2, XBP1, PPARGC1A, CLPP, and HNF1A. The altered expression levels of these genes contribute to the increased risk of tumor development.

Building on our understanding of PFOS and PFOA-induced cellular disruptions, key upstream regulators with abnormal expression patterns were further investigated. The top upstream regulators are PPARα, LARP1, ACOX1, MYC, MYCN, SLC27A2, XBP1, PPARGC1A, CLPP, and HNF1A. PPARα was the most upregulated gene, with its highest expression at a z-score of 9.684 in the mouse liver samples (Figure 8) [97]. It is involved in the regulation of lipid metabolism and inflammatory responses. The overexpression of PPARα contributes to increased fatty acid oxidation [98]. PPARα was also modulated in the downregulated LXR/RXR signaling pathway, as well as the upregulated fatty acid metabolism pathways (Figure 3). The LARP1 gene is another upstream regulator, and it showed downregulation across all samples (Figure 8). LARP1 regulates the translation of mRNAs and is involved in the eukaryotic translation pathway. When differentially expressed, it can alter oncogene expression (Figure 8) [99]. ACOX1 demonstrated a similar downregulation that contributes to the production of ROS as a byproduct of fatty acid oxidation (Figure 8). An increase in ROS is a hallmark of tumorigenesis and is associated with the upregulated pathways of oxidative phosphorylation and fatty acid beta oxidation 1 (Figure 6) [100]. The MYC and MYCN gene are both transcription factors and are involved with cell cycle progression. Their upregulation accelerates the cell cycle, which causes an increase in the expression of cyclins and CDKs and suppression of cell cycle inhibitors (Figure 8) [101]. Similar to PFOS and PFOA-exposed samples, the *MYC* gene was also upregulated in PFESA-BP2 (PFOS type) samples [102]. Its regulation was associated with increased CDK4 expression. The altered expression levels of these upstream regulators increase nutrient supply and contribute to tumorigenesis through disruptions in signaling pathways and cellular functions that fall under the key characteristics of carcinogens. Further analyses of additional upstream regulators and their potential roles in tumorigenesis are provided in Supplementary Section S3.

#### 3.3.7. Downstream Diseases and Functions Indicative of Tumor Initiation

**Figure 9:**
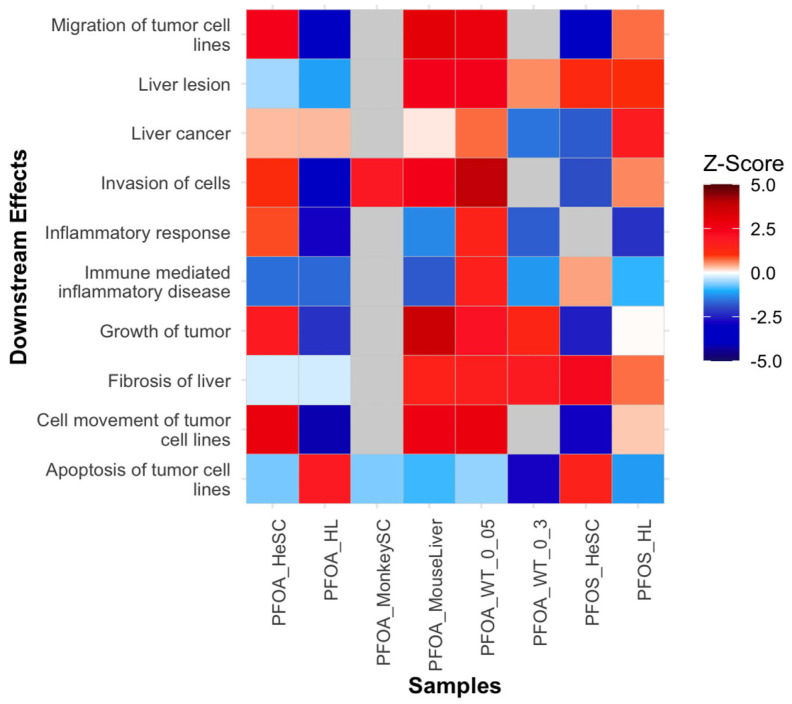
Top downstream diseases, biological, and toxicological functions and their predicted changes across all datasets. Red indicates predicted increased activity (positive z-score), while blue indicates predicted decreased activity (negative z-score) of that biological function or disease.

We also examined downstream biological functions, toxicological functions, and diseases of PFOS and PFOA-induced tumorigenic signatures. The analysis revealed diminished immune response of cells, immune-mediated inflammatory disease, and inflammatory response (Figure 9). This supports the PFOS/PFOA suppression of immune and inflammatory responses expanded upon in Section 3.3.3) that can promote a pro-tumorigenic environment. Additionally, the oxidation of lipids and liver lesions demonstrated enriched function. Concurrently, liver-associated pathologies including fibrosis of the liver, liver cholangiocarcinoma, and liver cancer were also enriched (Figure 9). The enrichment of such diseases and functions may suggest the potential development of HCC, which is further explained in Section 3.5.1. Notably, the processes of invasion of cells and growth of tumors were enriched, while apoptosis of tumor cell lines was diminished (Figure 9). These functions support the observed altered cellular proliferation induced by PFOS/PFOA (Section 3.3.2). These downstream effects identified collectively support that PFOS and PFOA exposure revealed an increase in tumor initiation pathways that are driven by altered immune response, oxidative damage, altered cellular proliferation, metabolic reprogramming, and epigenetic changes, as outlined above.

### 3.4. Evaluation of Concentration-Dependent Responses of PFOS and PFOA Exposure in Human Liver Samples

Concentration-dependent responses of PFOS and PFOA exposure were investigated in human liver samples (GSE144775) for distinct responses at high or low concentrations. Understanding the tumorigenic effects of PFOS and PFOA at varying concentrations is important for identifying early tumorigenic signatures and can help guide public health strategies for low or high concentration exposure.

#### 3.4.1. Differential Regulation of Liver Stress and Fibrosis at Low and High PFOA Concentrations

**Figure 10:**
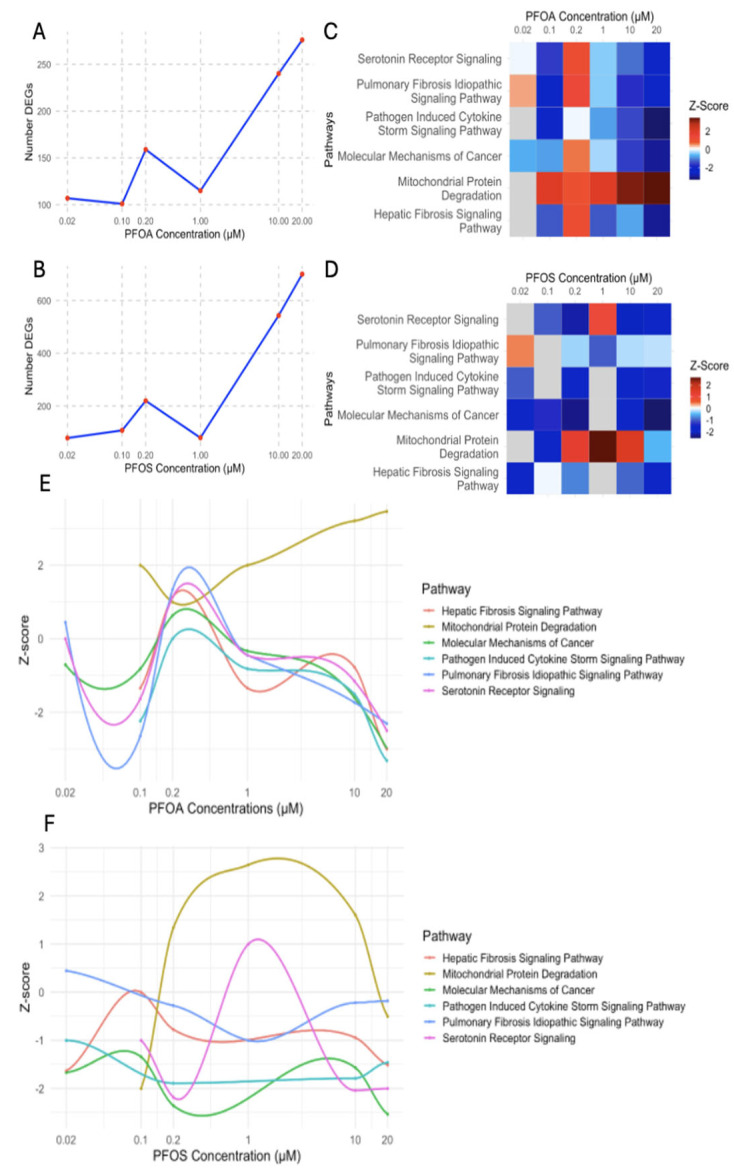
Concentration-dependent response analysis of PFOS and PFOA in human liver samples across six concentrations. (**A**) Number of DEGs present at each concentration of PFOS exposure. (**B**) Number of DEGs present at each concentration of PFOA exposure. (**C**) Heatmap of carcinogenic pathways and their z-score when exposed to PFOA. (**D**) Heatmap of carcinogenic pathways and their z-score when exposed to PFOS. Upregulation is indicated by red (positive z-score) and downregulation is indicated by blue (negative z-score). (**E**) Concentration response curve of carcinogenic pathways upon varying PFOA exposure. (**F**) Concentration response curve of carcinogenic pathways upon varying PFOS exposure.

The number of differentially expressed genes increased progressively with PFOA concentrations (Figure 10A). Notably, 0.2 µM conferred the strongest activation of tumor-promoting pathways, including hepatic fibrosis, pulmonary fibrosis, and molecular mechanisms of cancer (Figure 10C). This indicates PFOA-induced fibrosis and liver stress at low concentrations. Further, as the concentration increases, these pathways become downregulated, which shows a cellular attempt to suppress tumor-promoting processes (Figure 10C). In contrast, the mitochondrial protein degradation pathway showed progressive upregulation as the exposure concentration increased. Chronic activation of this pathway could impair mitochondrial function, a key factor in HCC development (Figure 10E). In all, PFOA exhibited a concentration-dependent response, where low concentrations of PFOA, especially 0.2 µM, activated tumorigenic pathways, while higher concentrations either amplified or inhibited these tumorigenic pathways.

#### 3.4.2. PFOS-Induced Nonlinear Regulation of Fibrosis, Immune, and Cancer Pathways

PFOS exposure demonstrates a more complex response with differing regulation patterns of pathways across concentrations. At 0.2 µM, PFOS induces a sharp increase in DEGs (220), but at 1 µM, the DEGs drop significantly (80) (Figure 10B). Interestingly, at 1µM concentration, there is also a marked decrease in pathway enrichment, where tumorigenic pathways are either differentially modulated or not affected (Figure 10D). At low concentrations, PFOS downregulated hepatic fibrosis signaling, molecular mechanisms of cancer, and serotonin signaling pathways. This represents an early disruption of liver repair mechanisms and fibrosis. The downregulation suggests that even minimal PFOS exposure can impair liver functions, which may promote the initiation of early stages of liver toxicity and HCC development (Figure 10D). At higher concentrations, PFOS exacerbated liver toxicity, evidenced by greater tumorigenic pathway z-scores (Figure 10D). At 20 µM, hepatic fibrosis signaling was strongly downregulated, which displays impaired liver repair and promotes fibrosis, a known precursor to HCC (Figure 10D) [103]. Similarly, molecular mechanisms of cancer were downregulated, which shows an attempt to suppress tumor-promoting processes (Figure 10D). Further, the pathogen-induced cytokine storm signaling pathway was downregulated across all concentrations of PFOS. Decreased cytokine storm signaling shows an impaired immune response and inflammation regulation (Figure 10F). In these findings, the concentration-dependent response of PFOS is nonlinear and warrants further investigation.

#### 3.4.3. PFOS and PFOA Exhibit Distinct and Shared Concentration-Dependent Mechanisms of Liver Toxicity

For both PFOS and PFOA, low concentrations can trigger significant transcriptional changes, while high concentrations induce higher amounts of DEG alterations. Both PFOS and PFOA reveal downregulation of key cancer pathways at higher concentrations. The downregulation of these pathways at higher concentrations could also suggest a potential suppression of carcinogenic processes at elevated concentrations (Figure 10C,D). Notably, the concentration-dependent effects of PFOS and PFOA differ at low concentrations. PFOS downregulated pathways related to liver repair and tumor suppression, while PFOA upregulated these pathways. PFOA strongly upregulated mitochondrial protein degradation, indicating enhanced mitochondrial activity, while PFOS showed no significant regulation of this pathway. These differences suggest that PFOS primarily exacerbates liver toxicity through impaired liver repair and immune suppression, while PFOA exposure can promote effects through mitochondrial dysfunction and oxidative stress. Furthermore, in both PFOS and PFOA, low concentrations are sufficient to exhibit tumorigenic signatures associated with initiation and development. These findings reveal PFOA exhibits a concentration-dependent response, in which lower concentrations activate tumorigenic pathways and higher concentrations downregulate these pathways. The concentration-dependent response of PFOS is variable.

### 3.5. PFOS and PFOA Potential Mechanisms Driving Tumorigenesis and Carcinogenesis

Biological pathways and markers were analyzed to hypothesize and propose potential mechanisms for different cancer types. First, we evaluated liver tumor initiation and development markers from the PFAS-exposed human and mouse liver samples. Next, we analyzed the prostate and testicular xenograft mouse tumor models for promotion and progression markers. Additionally, in the absence of kidney tissue data, we found conserved biomarker and pathway signatures that support initiation and development mechanisms of ccRCC. Based on these markers, we proposed hypothetical mechanisms of tumorigenesis and carcinogenesis. The mechanistic insights of this study should be interpreted as exploratory rather than definitive evidence of causality.

#### 3.5.1. PFOS and PFOA Exposure Increases Risk of HCC Initiation and Development

**Figure 11:**
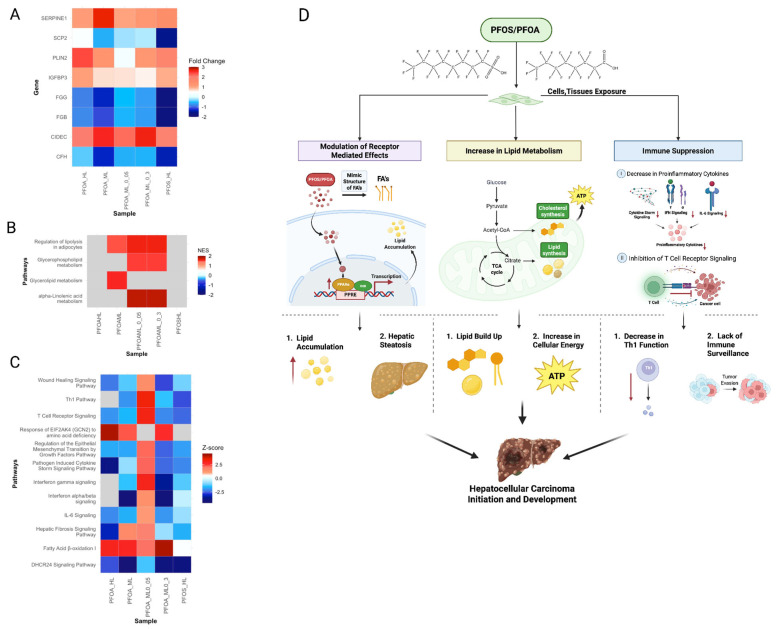
PFOS and PFOA disrupt lipid metabolism and induce inflammation contributing to HCC initiation and development. (**A**) Heatmap displaying fold changes in expression of liver disease-related biomarkers in response to PFOS and PFOA exposure. (**B**) GSEA pathway enrichment heatmap showing altered pathways following PFOA exposure. (**C**) IPA pathway heatmap demonstrating relevant pathway expression. (**D**) Summary of proposed mechanisms by which PFOS/PFOA exposure contributes to HCC development. PFOS/PFOA exposure modulates the activity of PPARα, which increases lipid accumulation, a precursor for hepatic steatosis. The upregulation of fatty acid beta oxidation 1 and other key lipid metabolism pathways result in a buildup of lipids and increase cellular energy. Additionally, the downregulation of pathogen-induced cytokine storm signaling, interferon gamma signaling, and IL-6 signaling suppresses immune cell function and overall immune activity. In parallel, inhibition of T cell receptor signaling prevents T cells from activating and developing their effector functions. These biological mechanisms may collectively drive HCC initiation and development.

PFOS and PFOA exposure upregulated lipid metabolism pathways such as fatty acid β-oxidation, glycerophospholipid biosynthesis, and mitochondrial dysfunction (Figure 11A–C). These alterations contribute to lipid accumulation and increase oxidative stress. This may lead to hepatic steatosis (Figure 11D) [104]. Further, as discussed in Section 3.3.1 PFOS and PFOA have the ability to mimic fatty acid structure and upregulate PPARα, thereby modulating lipid metabolism. One of the downstream targets of PPARα is *CIDEC*, a biomarker that was overexpressed in all liver samples (Figure 11D) [105]. This results in increased lipid droplets, which further demonstrates PFOS and PFOA disruption of lipid biosynthesis (Figure 11D). Such changes observed in lipid metabolism have been shown by Sangineto et al. to contribute to HCC initiation and development. Additionally, the downregulation of pathogen-induced cytokine storm signaling, IL-6 signaling, interferon gamma signaling, and other immune-related pathways suggest an immunosuppressive hepatic environment (Figure 11B–D). This immunosuppression facilitates an inflammatory state that may promote HCC development [106]. Furthermore, fibrosis of the liver was identified as a significantly upregulated downstream effect (Section 3.3.7). The concurrent dysregulation of T cell receptor signaling and hepatic fibrosis may reflect a fibrotic microenvironment due to a lack of immune surveillance. As shown by Zhang et al., such liver fibrosis modulates the activity of inflammatory cells and reduces natural killer T cell function. This disruption of inflammatory response is further demonstrated by the downregulation of *CFH* (Figure 11A). The absence of *CFH* can activate the complementary alternative pathway which contributes to chronic inflammation [107]. Moreover, the inhibition of *FGB* and *FGG* can further exacerbate liver fibrosis by impairing normal coagulation and tissue repair (Figure 11A) [108]. These altered processes collectively contribute to an inflammatory tumor microenvironment that may sustain HCC development (Figure 11D).

#### 3.5.2. PFOS Promotes Progression of Testicular Cancer

**Figure 12:**
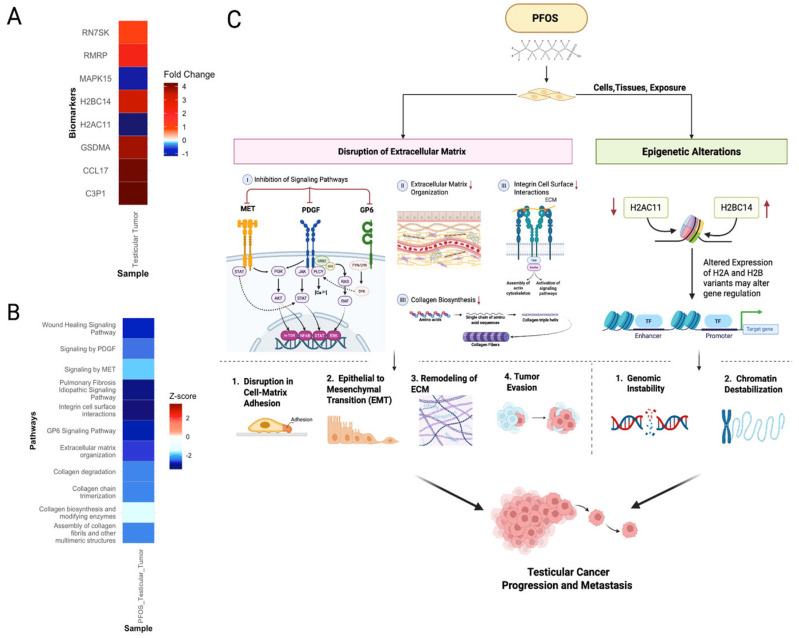
Inhibition of key signaling pathways, disruption of the ECM, and epigenetic modifications induced by PFOS exposure are potential mechanisms for testicular cancer progression. (**A**) Heatmap of significant biomarkers and differentially expressed genes involved in testicular cancer. (**B**) Heatmap of altered pathways induced by PFOS exposure. (**C**) Schematic demonstrating mechanisms associated with testicular cancer as a result of PFOS exposure. Inhibition of *MET*, *PDGF*, and *GP6* signaling pathways are shown to disrupt cell to matrix adhesion and allow for tumor evasion. Additionally, overexpression of *H2AC11* and *H2BC14* variants are depicted to potentially alter gene regulation and contribute to genomic instability as well as chromatin remodeling. The downregulation of extracellular matrix organization, integrin cell surface interactions, and collagen biosynthesis displayed lead to the remodeling of the ECM and epithelial to mesenchymal transition. These mechanisms potentially work collectively to progress and promote metastasis in testicular cancer.

PFOS-exposed testicular germ cell tumor samples exhibited downregulation of ECM organization, collagen biosynthesis, and integrin cell surface interactions pathways (Figure 12A,B). Such alterations may disrupt ECM structure and contribute to EMT, a process implicated in metastasis of testicular tumors [109]. Additionally, exposure to PFOS was associated with inhibition of *GP6*, *PDGF*, and *MET* signaling pathways (Figure 12B). Inhibition of these pathways reflects a disruption in normal cell–matrix adhesion and signaling, which facilitate tumor evasion (Figure 12C) [110]. The downregulation of wound healing and pulmonary fibrosis pathways suggest impaired tissue repair, a hallmark of aggressive tumors (Figure 12B) [111]. Furthermore, genes involved in chromatin remodeling and epigenetic regulation were highly upregulated (Figure 12A). The altered expression of *H2AC11* and *H2BC14* indicates disruption of nucleosome stability and histone variant composition (Figure 12C). These changes observed in H2A and H2B variants are associated with chromatin destabilization, genomic instability, and increased metastasis, as demonstrated by Lai et al. and Vardabasso et al. [112,113]. PFOS also upregulated *RMRP*, a long non-coding RNA associated with poor prognosis and malignant progression in various cancers (Figure 12A) [114]. Moreover, genes involved in immune modulation and inflammation, *C3P1*, *CCL17*, and *GSDMA*, were overexpressed (Figure 12A). These genes maintain T cell immunosuppression and chronic inflammation, promoting tumor immune escape and metastasis. Therefore, these findings suggest PFOS can promote testicular tumor progression and metastasis by disrupting ECM structure, epigenetic reprogramming, and immune evasion (Figure 12C).

#### 3.5.3. PFOS Drives Progression of Prostate Cancer

**Figure 13:**
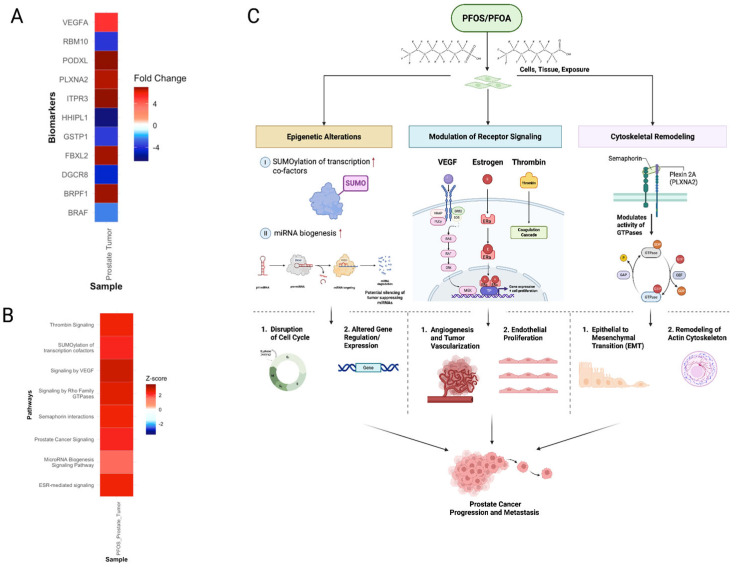
PFOS modulates receptor-mediated signaling and cytoskeletal organization to support the progression of prostate cancer. (**A**) Heatmap displaying differentially expressed biomarkers in PFOS-exposed prostate tumor samples and highlights genes involved in angiogenesis (VEGFA) and epigenetic regulation (*RBM10*, *BRPF1*). (**B**) Pathway enrichment analysis demonstrates the upregulation of oncogenic pathways in PFOS-exposed prostate tumors (**C**) Summary of the mechanistic effects of PFOS exposure on prostate cancer. PFOS modulates VEGF, ESR, and thrombin receptor-mediated signaling, and contributes to angiogenesis and endothelial cell proliferation. Additionally, upregulation of semaphorin signaling and *PLXNA2* (semaphorin receptor) alters the activity of rhoGTPases, which assists in the remodeling of the actin cytoskeleton. The upregulation of SUMOylation of transcription cofactors and miRNA biogenesis epigenetically impacts gene regulation and has the potential to disrupt the cell cycle. These molecular events collectively contribute to prostate cancer progression and metastasis.

PFOS exposure upregulated receptor-mediated signaling pathways of VEGF, estrogen receptor (ESR), and thrombin signaling (Figure 13A,B). VEGF and thrombin signaling increase endothelial proliferation and vascular permeability, which can enhance tumor vascularization (Figure 13C) [115,116]. Altered ESR signaling could further amplify this by promoting the transcription of pro-angiogenic genes (Figure 13B) [117]. ESR signaling is modulated by ERα, a receptor known to be directly bound and activated by PFASs. This suggests a potential mechanism for PFOS-induced dysregulation of hormone signaling in tumor progression (Figure 13C) [117]. These processes can work together or independently to promote the progression of prostate cancer through angiogenesis (Figure 13C). Moreover, PFOS exposure exhibited increased expression of epigenetic signatures such as the SUMOylation of transcription cofactors pathway and *BRPF1* gene (Figure 13A,B). SUMOylation is an epigenetic process active in rapidly proliferating tumor cells triggered by cellular stress, such as PFOS exposure. *BRPF1* is a chromatin regulator, which alters tumor-promoting transcriptional activity (Figure 13A). Further, the activation of the microRNA biogenesis pathway indicates post-transcriptional regulation disruptions, such as silencing of tumor-suppressive miRNAs (Figure 13B,C) [118]. Disruption of this pathway has been shown by Ramalho et al. to reduce invasion and increase apoptosis in prostate cancer cells (Figure 13C) [118]. Semaphorin signaling and its receptor *PLXNA2* were also upregulated, which suggests PFOS-induced cytoskeletal remodeling that may facilitate EMT and metastasis (Figure 13A–C) [119,120]. Additional biomarkers associated with prostate tumor progression include *PODXL*, *ITPR3*, and *FBXL2* (Figure 13A). *PODXL* upregulation promotes EMT and cellular invasiveness, while *ITPR3* enhances calcium signaling. These processes drive cell proliferation and resistance to apoptosis (Figure 13A) [121,122]. Downregulation of the tumor suppressor *FBXL2* impairs cell cycle control, allowing unchecked cell growth (Figure 13A) [123]. Collectively, these PFOS-induced signatures of receptor-mediated effects, epigenetic alterations, and cytoskeletal remodeling may establish an environment conducive to prostate cancer progression and promotion (Figure 13C).

#### 3.5.4. PFOS and PFOA-Induced Common Molecular Signatures of ccRCC Development

**Figure 14:**
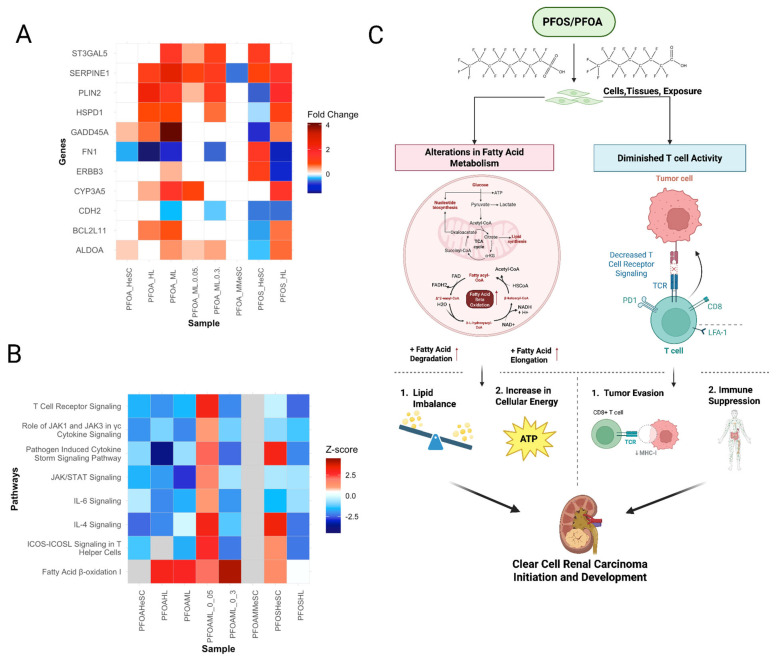
PFOS/PFOA exposure results in an alteration in fatty acid metabolism and diminished T cell activity. (**A**) Heatmap showing expression of ccRCC-associated genes following exposure to PFOA or PFOS. Genes such as *FN1*, *SERPINE1*, and *PLIN2* were upregulated under PFOS and PFOA exposure, consistent with tumorigenic pathways. (**B**) Heatmap of pathway enrichment demonstrates PFOS/PFOA-induced dysregulation of immune signaling and metabolic processes. (**C**) Illustration of proposed mechanisms by which PFOS/PFOA exposure contributes to ccRCC initiation and progression. PFOS/PFOA exposure upregulated fatty acid beta oxidation, fatty acid degradation, and fatty acid elongation. Upregulation of these pathways contributes to a lipid imbalance and increase in cellular energy. Furthermore, decreased T cell receptor signaling diminishes T cell activity, which allows for tumor evasion and decreases immune response. This decreased immune response contributes to overall immune suppression. Together, these processes have the potential to promote ccRCC initiation and development.

Our findings revealed pathway signatures and a significant amount of biomarkers (30%) in non-tumor samples associated with clear cell renal cell carcinoma (ccRCC) (Figure 14A). These include hallmark alterations in lipid synthesis through the upregulation of several fatty acid metabolism-related pathways (Figure 14B). Fatty acid degradation and elongation were both upregulated across almost all samples, demonstrating a lipid imbalance (Figure 14B,C). Additionally, both the synthesis and utilization of fatty acids were disrupted, which results in a buildup of lipid droplets and glycogen (Figure 14C) [124]. This is shown by the upregulation of glycerolipid metabolism, glycerophospholipid metabolism, linoleic/alpha-linoic lipid metabolism, and regulation of lipolysis in adipocytes (Figure 14B). Moreover, overexpression of *PLIN2*, a proposed prognostic marker in ccRCC, further supports the increased lipid metabolism in ccRCC (Figure S7; Section 3.3.1) (Figure 14A) [125]. Another key characteristic of ccRCC observed in our findings is an immunosuppressive microenvironment (Section 3.3.3). PFOS and PFOA exposure has been shown to induce oxidative stress within immune cells and downregulate inflammatory pathways, which impairs immune cell function in such environments. The decrease in immune response and T cell activity further indicate a lack of immune surveillance (Figure 14C) [126]. In parallel, *SERPINE1*, a key overexpressed prognostic biomarker, increases immune cell infiltration and upregulates immunosuppressive effects to contribute to an active tumor microenvironment (Figure 14A; Figure S3) [127,128]. These results show that PFOS and PFOA are associated with an increased cell metabolism and impaired immune function, which increases the chance of ccRCC tumorigenesis (Figure 14C) [124].

### 3.6. Extrapolation of Tumorigenic Signatures from PFOS-Exposed Non-Tumor to Tumor Samples

**Figure 15:**
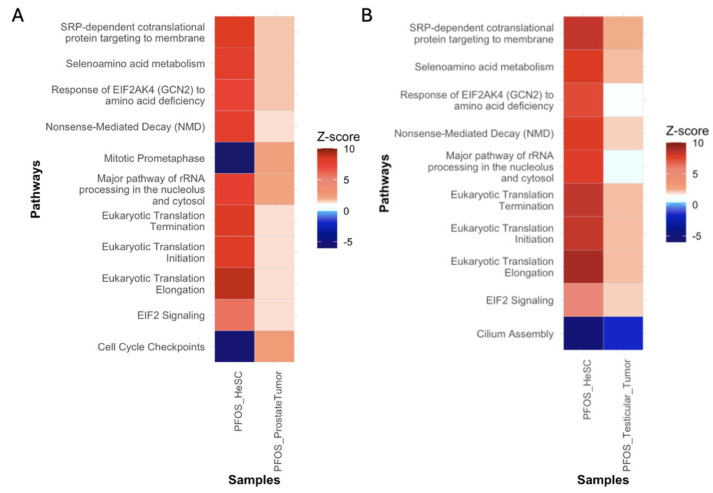
Comparison of PFOS-exposed non-tumor and tumor signatures. Heatmap of z-scores of the top pathways of PFOS-exposed human embryonic stem cells and the xenograft tumor models. Red indicates a positive z-score, blue indicates a negative z-score. (**A**) Prostate tumor and non-tumor comparison. (**B**) Testicular tumor and non-tumor comparison.

We conducted an extrapolation of tumorigenic signatures in PFOS-exposed non-tumor to tumor samples to determine signatures in normal tissues that contribute to or persist in tumorigenic states. To extrapolate, we compared the IPA pathways of the human embryonic stem cells with the testicular tumor and prostate tumor samples. The PFOS-exposed non-tumor human embryonic stem cells had notable overlap with the prostate and testicular tumor samples in pathways of protein synthesis, protein translation, and ER stress. Pathways included eukaryotic translation initiation, elongation, termination and SRP-dependent cotranslational targeting pathways (Figure 15A,B). These findings build upon Section 3.3.2 where upregulated protein synthesis and amino acid metabolism were identified as responses to PFOS and PFOA in non-tumor samples. Notably, both tumor comparisons exhibited increased EIF2 signaling and EIF2AK4 response to amino acid deficiency (Figure 15A,B). These pathways regulate stress response and are activated in response to ER stress [129]. This consistent upregulation of EIF2 signaling in non-tumor and tumor samples suggests that PFOS-induced ER stress may serve as a shared initiator and promoter of prostate and testicular tumorigenesis. In prostate cancer, overactivation of EIF2 signaling in tumor cells is associated with cancer cell survival, stress adaptation, and progression, as shown by Li et al. [130]. Additionally, Karna et al. demonstrated ER stress in testicular tissue may contribute to testicular tumorigenesis by triggering cell death and disrupting protein homeostasis [131]. While Section 3.3.2 discussed the role of increased protein translation and amino acid metabolism in creating a pro-tumorigenic state, the extrapolation to tumor samples demonstrate that these signatures are retained and potentially amplified in tumor samples, which can contribute to both testicular and prostate tumor initiation and maintenance.

### 3.7. Identification of Novel Therapeutic Targets as a Rescue Strategy: Prevention and Treatment Strategy

Inspired by our earlier drug repurposing studies, we investigated therapeutic strategies, guided by the molecular markers and signatures identified in this study, that can rescue the potential tumorigenic effects of PFOS and PFOA exposure [132,133,134,135,136]. Non-tumor samples were analyzed to propose preventative strategies, such as chemopreventive drugs, that may reduce the risk of tumor initiation. Tumor samples were used to evaluate treatment strategies for reversing or suppressing tumor-promoting mechanisms. This therapeutic analysis, including both biomarker targets and mechanistic pathways, enabled the identification of potential interventions for both prevention and treatment of PFOS and PFOA-induced tumorigenic effects. While the identification of these therapeutic targets and strategies provides valuable insights into the translational potential of our findings, we acknowledge that these results are preliminary and require direct experimental validation.

From the identified biomarkers, we selected potential therapeutic targets based on druggability, overall fold change across datasets, and known effects on diseases or symptoms correlated with PFOS/PFOA exposure (Section 3.2). Genes were classified as oncogenes or tumor suppressors to identify carcinogenic target potential. Therapeutic potential of these biomarkers was assessed based on drug–gene interactions as well as current target use in treatment for cancer and other related disorders. A drug–gene interaction is classified as an interaction (such as inhibition) between a known drug compound and an interacting gene during the drug’s mechanism of action. In addition to drug–gene interactions, genes were further assessed to determine whether they are current targets of any drugs to examine the therapeutic use. Although these genes require further analysis, we propose these potential genes and their corresponding proteins as potential therapeutic targets to rescue the carcinogenic and toxic effects of PFOS/PFOA exposure based on their functionality, previous therapeutic uses, and drug–gene interactions. In parallel, the identified tumorigenic mechanisms of PFOS and PFOA were examined for their therapeutic potential (Section 3.5). The upstream and downstream molecular effectors that regulate these tumorigenic and carcinogenic signatures were evaluated as potential mechanistic targets. These targets were cross-referenced with known drug modulators reported in the literature to assess their potential for therapeutic intervention. These prevention and treatment strategies identified are discussed in the following sections.

#### 3.7.1. Preventative Interventions for Cancer Initiation

Out of the 30 biomarkers from our comprehensive biomarker profile, genes with high drug–gene interaction scores and cancer association were selected for further analysis (Section 3.2). Table 4 presents the biomarkers with strong therapeutic relevance and drug candidates that may modulate early PFOS and PFOA-induced tumorigenic changes. Notably, *SERPINE1* and *FN1* were identified as potential markers of tumor initiation, with multiple drug–gene interactions. *SERPINE1* upregulation was associated with pro-angiogenic and immunosuppressive activity (Section 3.3.3) and is targeted by compounds such as urokinase and epirubicin. *FN1* downregulation can be indicative of ECM degradation, which can be restored by ECM-modifying agents such as Ocriplasmin. Additional targets including *ALDOA*, *PLIN2*, and *TRIB3* were upregulated, reinforcing metabolic dysregulation as early tumorigenic mechanisms of PFOS and PFOA. Drugs proposed such as Zinc Acetate, Dihydroxyacetone Phosphate, and RTI-122 can target these genes that modulate the altered metabolic reprogramming to prevent tumorigenic transformation. Additionally, *NQO1* and *CDH2* were consistently downregulated tumor suppressor genes across PFOS and PFOA-exposed samples. The restoring function of these TSGs can reverse the tumorigenic processes they are involved in. *NQO1* is involved in redox balance, but when they are downregulated, they cannot respond to increased oxidative stress, which is a consistently enriched process in PFOS and PFOA-exposed samples (Section 3.3.4). Vitamin E, cannabidiol, and carboplatin can increase *NQO1* and similar defenses against oxidative stress. Similarly, loss of *CDH2* function can disrupt EMT and cell–cell adhesion; thus, targeting this gene with Methadone can suppress this tumorigenic process (Section 3.3.2).

Due to the bioaccumulative nature and tumorigenic potential of PFOS/PFOA in the body, alternative strategies for mitigating the adverse effects of PFOS and PFOA are necessary. We identified several natural compounds and dietary interventions that target PFOS/PFOA-induced mechanisms of tumorigenicity that may prevent or alleviate the effects of exposure to PFASs (Table 5). Kaemferol and omega-3 PUFAs target PPARa and reduce lipid accumulation, reversing metabolic and inflammatory reprogramming induced by PFAS exposure (Section 3.3.1). Glutathione and vitamin C can protect against ROS accumulation and fibrosis (Section 3.3.4). These dietary/natural-based therapeutics are more accessible approaches to counteracting PFOS/PFOA-induced carcinogenic mechanisms.

#### 3.7.2. Treatment Strategies for Prostate and Testicular Tumor Progression

The biomarkers with the highest drug–gene interaction scores from the prostate tumor and testicular tumor samples were evaluated for therapeutic potential (Section 3.5.2 and Section 3.5.3). Among the shared targets in prostate and testicular tumors, *HSPA5*, *CDH11*, and *GSTP1* were identified as key carcinogenic markers (Table 6). Targeting these genes can help reduce PFOS-induced ER stress, deactivation of tumor suppressor genes, and genomic instability [142,143,144]. Notably, our previous study identified several potential *CDH11* small molecule inhibitors, including SD-133, FDA approved celecoxib, and DMC (dimethyl celecoxib) [142,145]. These compounds may exert their anticancer effects through modulation of *CDH11*-associated signaling pathways. Celecoxib and DMC, in particular, are known *COX-2* inhibitors but also have been shown to interact with cadherin-mediated cell adhesion pathways. Thus, these drugs can be repurposed as a targeted rescue strategy in prostate and testicular tumors [142,145]. By inhibiting *CDH11*, these agents may not only reduce cell proliferation and metastasis but also mitigate PFOS-induced testicular and prostate tumor-promoting mechanisms such as inflammation and EMT.

Similarly, overexpression of *FGF10* in PFOS-exposed testicular tumors is associated with EMT and tumor progression. Clinically approved FGFR inhibitors, such as infigratinib and pemigatinib, may counter *FGF10* signaling to reverse testicular tumor progression (Section 3.5.2). In prostate cancer specifically, *GSTP1*, involved in xenobiotic detoxification and chemoresistance, is a viable target for small molecule inhibitors. These inhibitors, such as Canfosfamide, may decrease *GSTP1*’s enzymatic function to reduce the detoxification of chemotherapeutic agents [143,146]. Further drug interaction data from DGIdb and DrugBank used to identify existing compounds that either interact with or directly target such genes, biomarkers, and existing drug candidates are presented in Table 6.

We proposed mechanistic-based therapeutic interventions aimed at reversing the PFOS-induced carcinogenic processes hypothesized in earlier sections (Table 7) (Section 3.5.2 and Section 3.5.3). Prostate and testicular tumor samples revealed shared mechanisms of tumor promotion, including ER stress and EIF2a signaling (Section 3.6). Clinically tested compounds such as ISRIB and PERK inhibitors have shown efficacy in interrupting these pathways to slow and/or reverse tumor promotion. In prostate tumors, PFOS upregulated VEGF signaling, thus increasing angiogenesis, a hallmark of tumor growth (Section 3.5.3). Anti-angiogenic agents such as Bevacizumab can target VEGF-A to limit tumor vascularization [147]. Similarly, therapeutic inhibition of *VEGFR1* and *VEGFR2* may also offer strategies to suppress tumor vascularization. Earlier, we identified isoindole-based inhibitors as small molecule VEGFR antagonists that can selectively block VEGF-mediated signaling and angiogenesis in solid tumors [148]. This can be repurposed to potentially mitigate PFOS-induced prostate tumor progression (Section 3.5.3). PFOS also activated ESR signaling in prostate tumors, which can be antagonized by Toremifene to reduce hormone tumor growth (Section 3.5.3). For testicular tumors, Pirfenidone is proposed to target PFOS-induced ECM remodeling and fibrosis to limit metastasis (Section 3.5.2).

## 4. Strengths

To the best of our knowledge, this is the first comprehensive multi-organ, cross-species study to investigate the mechanisms of PFOS- and PFOA-induced carcinogenesis by integrating pathway, biomarker, and upstream and downstream regulator analyses within a structured framework. A major strength of this work is the development of a reproducible RStudio pipeline for RNA sequencing, gene set enrichment, concentration-dependent response, and *p*-value combination meta-analysis. This workflow was validated against published datasets to confirm accuracy and reliability, and the results were further reinforced by a *p*-value combination meta-analysis, ensuring robustness of the conserved transcriptomic signatures. The RNA-sequencing analysis pipeline was validated against published datasets, confirming its accuracy and reliability. Additionally, the mechanisms proposed in this paper follow the key characteristics of the carcinogen framework established by the IARC. This structured approach allowed analysis of consistent mechanistic patterns across the various samples. Our method of extracting and categorizing biomarkers from existing datasets has proved to be both accurate and coherent. Both biomarkers and canonical pathway regulation in PFOS/PFOA-exposed tissues generally followed similar trends across similar, independent datasets. The biomarkers we extracted and analyzed were also coherent with the upregulated or downregulated canonical pathways we extracted, generally matching experimental results from previous publications. In general, our procedure in categorizing PFOS/PFOA-caused cancer-related biomarkers has provided more general information on the effects of PFOS/PFOA on cancer risk and carcinogenesis, which was an area of study that was not previously well-explored. Finally, we established a therapeutic target identification pipeline by integrating DEG data with drug–gene interaction databases (DrugBank, DGIdb). Further, we proposed natural compounds as accessible chemopreventive agents. By linking the observed transcriptomic alterations to accessible therapeutic strategies, this study provides translational value for development of therapeutic treatment and disease prevention methods.

## 5. Limitations

While our study provides promising information regarding PFOS/PFAS exposure to cancer risk, our study has limitations that should be acknowledged. Firstly, this study integrates datasets across species, tissues, and exposure paradigms, which raises the possibility of heterogeneity and potential batch effects. To mitigate these effects, we analyzed each dataset independently with a standardized DESeq2 workflow, applied consistent normalization, and used correlation analyses to verify that conserved responses. We further conducted a *p*-value combination meta-analysis, which accounts for inter-study variability, and it was consistent with the independent DeSeq2 analyses strengthening confidence in conserved responses identified. Nonetheless, differences in tissues, species, exposure paradigms, and experimental design remain potential sources of bias. Additionally, the scope of this study is transcriptomic and therefore restricted to gene expression. While differential gene expression and pathway enrichment can reveal signatures consistent with carcinogenic mechanisms, the signatures are correlative rather than casual. Our results provide strong rationale for PFOS/PFOA-driven tumorigenic mechanisms; however, complementary validation at proteomic, epigenomic, and function levels is required to confirm their biological and clinical relevance. While the pathway and biomarker signatures provide strong rationale for potential mechanisms of PFOS/PFOA-driven tumorigenesis, future studies as well as experimental validation will be essential to confirm these mechanisms Although there is an absence of kidney samples, we hypothesized mechanistic conclusions for ccRCC from the observed disruptions in DEGs and non-tumor pathways. Additionally, mechanistic hypotheses for ccRCC, prostate tumors, and testicular tumors remain speculative given the limited datasets and use of xenograft models. Finally, although we highlight putative therapeutic targets and chemopreventive compounds, these remain predictive. Validation in controlled in vitro and in vivo models is warranted to establish their functional and clinical relevance. Accordingly, all our findings should be viewed as hypotheses and will require validation in controlled experimental systems. Acknowledging these limitations is essential for the whole interpretation of our study’s findings and for guiding further research endeavors in this field.

## 6. Conclusions

This study provides novel insights into the potential molecular mechanisms by which PFOS and PFOA exposure contributes to tumorigenesis and carcinogenesis. Our results show that PFOS and PFOA exposure consistently disrupted seven key characteristics of carcinogens. The comprehensive biomarker profile consists of key biomarkers which can serve as early signatures of PFOS and PFOA-induced tumorigenesis involved in lipid metabolism, ECM remodeling, immune evasion, and oxidative stress. Immune and inflammatory signaling pathways were broadly downregulated, suggesting impaired immune surveillance and a tumor-permissive microenvironment. In parallel, oxidative stress and mitochondrial pathways were upregulated, supporting sustained ROS generation and mitochondrial stress, indicative of oxidative damage. Furthermore, epigenetic alteration signatures were reflective of genomic instability and silencing of tumor suppressors. The overexpression of PPARa combined with the consistent upregulation of lipid metabolism pathways and related DEGs suggests lipid metabolic disruption as a central mechanism. Additionally, we found that even low, environmentally relevant, concentrations of PFOS and PFOA are sufficient to initiate tumorigenic signatures in human liver samples. These results provide evidence to guide public health regulatory policies. Our findings suggest potential PFOS and PFOA-associated tumorigenic mechanisms such as lipid metabolic disruption in HCC, receptor-mediated signaling in prostate cancer, epigenetic instability and ECM remodeling in testicular tumors, and immunosuppression in ccRCC. Lastly, therapeutic analysis identified multiple druggable targets and natural chemopreventive compounds that may mitigate the negative effects of PFOS and PFOA exposure.

By integrating pathway analyses with biomarker identification and therapeutic target discovery, our findings contribute to the growing understanding of PFAS-induced tumorigenesis. All our findings should be viewed as hypotheses and additional research is needed to further validate these mechanisms of tumorigenicity, biomarkers, and therapeutic targets, and to assess the clinical relevance of these findings for cancer therapy.

## Figures and Tables

**Table 1:** Summary of PFOS and PFOA-exposed cross-tissue and species datasets utilized in this study.

GEO ID	Samples	PFOS and PFOA + Control Samples (Total 529)	PFAS Type	Organism	Cell Line/ Tissue Type	PFOS/PFOA Concentration	Reference
GSE236956	42	18	PFOS, PFOA	Homo sapiens	Embryonic stem cells hESC H1 line from the National Stem Cell Bank ℅ the WiCell Research Institute Differentiated into non-neuroectoderm (NNE) cells	10 μM	Zhao et al., 2024 [25]
GSE144775	607	406	PFOS, PFOA	Homo sapiens	Human liver spheroids 3D InSight Human Liver Microtissues from InSphero. Spheroids from this manufacturer are a co-culture model pooled from 10 different human liver donors.	0.02, 0.1, 0.2, 1, 2, 10, 20, 50, 100 µM	Rowan-Carroll et al., 2021 [26]
GSE262137	26	26	PFOS	Homo sapiens	Testicular germ cell tumor cells Human embryonal carcinoma cell lines 2102EP and NT2/D1 from ATCC Xenografted into immunocompromised athymic nude mice	5 µM	Boyd et al., 2024 [27]
GSE185183	23	23	PFOS	Mus musculus	Prostate cancer xenograft Congenic RWPE-1 (non-tumorigenic) and RWPE-kRAS (tumorigenic w/K-ras oncogene transfection) from ATCC. Xenografted 2 × 10^6^ RWPE-kRAS cells into athymic nude male mice after HFD treatment.	10 mg/kg/d Cells were treated with PFOS at 10 µM, 1 µM, 0.1 µM, 0.01 µM, 0.001 µM, 0.0001 µM, with or without 0.001 µM DHT, before xenograft.	Imir et al., 2021 [28]
GSE119441	16	16	PFOA	Mus musculus	Mouse liver cells Mice genotype: C57BL/6	1 mg/kg/ bw/day	Li et al., 2019 [29]
GSE212294	32	24	PFOA	Mus musculus	Mouse liver cells in C57BL/6 wildtype and PPARα knockout mice	0.05 mg/kg bw/day 0.3 mg/kg bw/day	Attema et al., 2022 [30]
GSE86939	29	10	PFOA	Macaca mulatta	Embryonic stem cells Rhesus embryonic stem cells (ESCs) Line: Oregon Rhesus Macaque Embryonic Stem (ORMES)-6, 42XX from the Oregon National Primate Research Center	0.1 µM	Midic et al., 2016 [31]

**Table 2:** Validation of RNA-sequencing workflow using the GSE236956 dataset.

	RStudio DeSeq2 Analysis			Experimental Value			Difference Between R Sequencing and Published Data		
Gene Name	log_2_FoldChange	*p*-Value	FDR	log_2_FoldChange	*p*-Value	FDR	log_2_FoldChange	*p*-Value	FDR
CEP131	−0.171	0.239	0.486	−0.171	0.243	0.553	0	−0.004	−0.067
EGFR	0.857	2.60 × 10^−18^	1.49 × 10^−16^	0.857	2.11 × 10^−18^	1.36 × 10^−16^	0	4.90 × 10^−19^	1.30 × 10^−17^
A1BG	−0.017	0.956	0.985	−0.018	0.954	0.993	0.001	0.002	−0.008
SOX7	−0.55	0.169	0.391	−0.548	0.175	0.452	−0.002	−0.006	−0.061
GPC5	1.141	0.016	0.067	1.142	0.018	0.086	−0.001	−0.002	−0.019

**Table 3:** Key characteristics of carcinogens displayed in PFOS and PFOA-exposed samples.

Key Characteristics	PFOS	PFOA	Pathways Evidence Across PFOS/PFOA-Exposed Datasets	Biomarkers
1. Is electrophilic or can be metabolically activated	No Data	No Data	No Data	No Data
2. Is genotoxic	No Data	No Data	No Data	No Data
3. Alters DNA repair or causes genomic instability	Upregulated	Upregulated	Disruption of pathways crucial for maintaining DNA integrity, proper cell cycle progression, and accurate protein synthesis promote DNA damage and genomic instability	*XBP1*, *GADD45a*
4. Induced epigenetic alterations	Upregulated	Upregulated	Disruption of pathways that impair DNA methylation, chromatin remodeling, and histone modifications, silencing tumor suppressor genes and promoting epigenetic modifications	*FOXO3*
5. Induces oxidative stress	Upregulated	Upregulated	Upregulation of genes involved in oxidative phosphorylation, fatty B-oxidation, and mitochondrial dysfunction that are associated with ROS production, implicating PFOS and PFOA exposure with inducing oxidative stress	*CYP3A5*, *ALDOA*, *SERPINE1*, *NQO1*, *HSPD1*, *PLIN2*, *ALDH3A2*
6. Induces chronic inflammation	Downregulated	Downregulated	Downregulation of inflammatory pathways that resolve inflammation, indicating PFOS and PFOA exposure can lead to a state of chronic inflammation	*FN1*, *ID1*, *HSPD1*, *TRIB3*, *SESN2*
7. Is immunosuppressive	Downregulated	Downregulated	Suppression of immune surveillance and elimination of potentially malignant cell pathways, PFOS and PFOA may act as carcinogen by impairing immune function	*ID1*, *HSPD1*, *TSC22D3*
8. Modulates receptor-mediated effects	Upregulated	Upregulated	Upregulation of lipid metabolism and metabolic reprogramming pathways suggests PFOS and PFOA can act as ligands for carcinogenic pathways	*ALDOA*, *PLIN2*, *IGF1*, *LDLR*
9. Causes immortalization	No Data	No Data	No Data	No Data
10. Alters cell proliferation, cell death, or nutrient supply	Upregulated	Upregulated	Upregulation of pathways involved in protein synthesis, metabolic reprogramming, and ECM organization indicates disruption in cell proliferation, death, and nutrient supply	*BCL2L11*, *PSAT1*, *SERPINE1*, *CDH2*, *EIF4EBP1*, *ERBB3*, *GLUL*, *COMT*, *IGF1*, *RPL9*, *RPS6*, *TRIB3*, *LAMP3*, *HSD17B11*, *ATF4*, *P4HA2*, *NUCB2*, *SDC1*, *ACTA1*

**Table 4:** Potential therapeutic targets for PFOS/PFOA exposure identified based on therapeutically classified biomarkers, functionality, druggability, and previous uses.

Gene	Oncogene or Tumor Suppressor Gene	Basic Functionality	Therapeutic Uses Based on Drug–Gene Interactions	Drugs that Interact with Gene Based on DGIdb	Drugs with Gene as a Target Based on DrugBank
*SERPINE1*	N/A	Plasminogen-activator inhibitor, primary inhibitor of proteins that breaks down blood clots	Used in thrombotic and antithrombotic therapy as well as an antineoplastic agents	Epirubicin, Urokinase, Arsenic Trioxide, Cetrorelix	Drotrecogin Alfa, Urokinase, Troglitazone, Alteplase, Anistreplase, Reteplase, Copper
*ALDOA*	Oncogene	Key enzyme in glycolysis pathway	Therapeutic use in metabolic disorders	Propionic Acid, Acetate	Dihydroxyacetone Phosphate, Zinc, Artenimol, Zinc Acetate, Zinc Chloride, Zinc Sulfate
*FOXO3*	Oncogene	Transcription factor involved in cell cycle regulation, apoptosis, and oxidative stress response	Targeted in cancer therapy and longevity research	Resveratrol, Syringaresinol	None identified
*IGFBP3*	N/A	Carrier protein for insulin-like growth factor signaling	Therapeutic use in palliative chemotherapy for gastric cancer	Fluorouracil, Celecoxib	Mecasermin, Alitretinoin
*FN1*	N/A	Responsible for cell adhesion and migration due to role in ECM	Antineoplastic therapeutic use in chemotherapy	Dacarbazine, Ocriplasmin	Ocriplasmin, Lanoteplase, Zinc, Zinc Acetate, Zinc Chloride, Zinc Sulfate
*NQO1*	Tumor Suppressor Gene	Antioxidant enzyme regulating redox balance, detoxification, and tumor suppression	Antioxidant defense, chemotherapy activation, tumor suppression, and proteasome regulation	Itraconazole, Dicumarol, Benzene	alpha-Tocopherol succinate, Cannabidiol, Carboplatin, Cisplatin, D-alpha-Tocopheryl acetate, Vitamin E, Flavin adenine dinucleotide, Menadione, Oxaliplatin, Phenytoin
*CDH2*	Tumor Suppressor Gene	Encodes N-cadherin, involved in cell–cell adhesion and EMT	Targeted in cancer therapy, particularly metastasis inhibition	Methadone, hydrochloride Adh1	None identified
*ERBB3*	Oncogene	Member of the EGFR family, involved in cell growth and differentiation Targeted in cancer therapy for inhibition of signaling pathways	Targeted in cancer therapy for inhibition of signaling pathways	AV-203	Patritumab, LJM716, Duligotuzumab
*PLIN2*	N/A	Promotes formation in lipid droplets resulting in lipid accumulation	Therapeutic use in NAFLD and metabolic disorders	RTI-122	No current drugs using gene as a target

**Table 5:** Dietary and natural therapeutic interventions to oppose potential carcinogenic effects of PFOS/PFOA exposure.

PFOS/PFOA Mechanism	Target	Compound	Source	Reasoning	Therapeutic Benefit
PPARα upregulation + lipid metabolism dysregulation	PPARα	Kaempferol [137]	flavonoid found in plant-based foods, including green tea, broccoli, kale, and berries	Kaempferol stably binds to PPARα restoring lipid homeostasis and exhibit anticancer properties	Reduces PFOS/PFOA-induced lipid dysregulation and potentially mitigates tumorigenic effects
Lipid accumulation and inflammation	PPARs	n-3 PUFAs: (eicosapentaenoic acid (EPA) and docosahexaenoic acid (DHA)) [138]	Found in fish oil, flaxseeds, and walnuts	N-3 PUFAs activate PPARs, restoring PPAR signaling to mitigate lipid accumulation and inflammation	Counteracts PFOS/PFOA-induced metabolic dysregulation and inflammation
ECM remodeling and hypoxia	ECM proteins and HIF-1	Vitamin C [139,140]	Found in citrus fruits, bell peppers, and leafy greens	Vitamin C promotes ECM repair and inhibits HIF-1 activation	May prevent fibrosis and hypoxia-driven tumor progression, driven by dysregulation of FN1 and PLIN2 genes, induced by PFOS/PFOA exposure
Oxidative and ER stress	Antioxidant response genes	Glutathione [141]	Endogenously synthesized; also found in spinach, avocados, and asparagus	Glutathione increases antioxidant capacity and reduces ER stress	Protects against oxidative damage and cellular stress responses triggered by PFOS/PFOA

**Table 6:** Therapeutic targets for PFOS-exposed prostate and testicular tumor samples.

Gene	Basic Functionality	Therapeutic Uses Based on Drug–Gene Interactions	Drugs That Interact with Gene Based on DGIdb	Drugs with Gene as a Target Based on DrugBank	Prostate/Testicular
*HSPA5*	RNA binding function, regulates calcium, and responds to ER stress	Inhibition of HSPA5 assists in managing ER stress and protein abundance	Mianserin	No interactions found	Prostate
*CDH11*	Regulates cell adhesion-known tumor suppressor gene	Antibodies can target overexpressed CDH11 to inhibit tumor progression	No interactions found	Celecoxib	Prostate
*GSTP1*	Detoxifies foreign substances, preserves DNA and prevents damage to DNA	Under hypoxic conditions where GSTP1 is overexpressed, inhibitors can reduce resistance to therapy and metastasis	Cytarabine Docetaxel Anhydrous Daunoribicin Liposomal	Canfosfamide 2-(N-morpholino)ethanesulfonic acid Cibacron Blue Clomipramine Clozapine Curcumin	Prostate
*FGF10*	Growth factor involved in EMT and tissue repair	FGFR signaling inhibitors are used in tumors to inhibit EMT and tumor progression	No interactions found	Erdafitinib, Futibatinib, Infigratinib, Lenvatinib, Nintedanib, Palifermin, Pemigatinib, Regorafenib	Testicular
*HMGB1*	Involved in replication, transcription, chromatin remodeling, V(D)J recombination, DNA repair and genome stability	Antagonists can inhibit HMGB1 by interfering with the cytoplasmic function. Inhibition of HMGB1 decreases inflammation	Itraconazole Prednisolone	Chloroquine Ethyl pyruvate	Prostate and Testicular

**Table 7:** Therapeutic targets for PFOS-exposed prostate and testicular tumor samples based on proposed carcinogenic mechanisms.

Mechanism	Target	Reasoning	Cancer Type Targeted	Drug/Compound	Drug Status	Mechanism of Action
Overactive estrogen receptor signaling	ERα	PFOS promotes Erα-mediated transcription, which supports hormone-driven tumor progression	Prostate	Toremifene (nano-targeted delivery) [149]	Chemopreventive (off-label); FDA approved for metastatic breast cancer [150]	Selective estrogen receptor modulator (SERM); antagonizes ERα and downregulates estrogen responsive genes
VEGF promoted angiogenesis	VEGF-A	PFOS upregulates VEGF signaling, which enhances tumor vascularization and angiogenic signaling	Prostate	Bevacizumab	FDA-approved for several cancers	Humanized monoclonal antibody targeting VEGF-A; inhibits angiogenesis
VEGF promoted angiogenesis	VEGFR1/2	VEGFR1/2 activation promotes angiogenesis and metastasis in tumors; inhibition can suppress PFOS-induced vascular remodeling	Prostate	Isoindole-based inhibitors (VGA1102, VGA1155)	Experimental, patented small molecules	Small molecule inhibitors of VEGFR1 and VEGFR2; block VEGF binding and signaling
Fibrosis associated progression	TGF-β and collagen synthesis	PFOS alters ECM and fibrosis-related pathway which promotes pro-tumorigenic microenvironment	Testicular	Pirfenidone [151]	FDA-approved for idiopathic pulmonary fibrosis	Anti-fibrotic; inhibits TGF-β signaling and collagen production
ER stress	PERK (EIF2AK3)	PFOS exposure induces ER stress which initiates the UPR via PERK. This promotes cell survival and contributes to tumor maintenance under ER stress	Testicular and Prostate	GSK2606414 or GSK2656157 [152]	Experimental	PERK inhibitors: bind to PERK kinase domain to prevent EIF2α phosphorylation
EIF2 signaling	EIF2α (EIF2S1)	PFOS upregulated EIF2 phosphorylation in non-tumor and tumor samples. This suppresses translation while enhancing stress response	Testicular and Prostate	ISRIB (Integrated Stress Response Inhibitor)	Experimental	Allosteric activator of eIF2B that restores protein translation despite phosphorylated EIF2α

## Data Availability

All the RStudio codes used for RNA sequencing, molecular signature analysis, and figure generation are available on Github at the following address: https://github.com/sivaGU/PFOS-PFOA-Exposure-Analysis (accessed on 7 August 2025).

## Supplementary Materials

The following supporting information can be downloaded at: https://www.mdpi.com/article/10.3390/cimb47090763/s1.
