# Supplementary material for: Uncovering the Tumorigenic Blueprint of PFOS and PFOA Through Multi-Organ Transcriptomic Analysis of Biomarkers, Mechanisms, and Therapeutic Targets
Source: Curr Issues Mol Biol. 2025 Sep 15;47(9):763. doi: 10.3390/cimb47090763 (PMC12468109; doi:10.3390/cimb47090763)
Supplement: Supplementary file 1 [file cimb-47-00763-s001.zip › Supplementary Document.pdf]

Legend

Section S1: Batch Effect Assessment and Meta-Analysis Validation

Figure S1: Correlation plot and meta-analysis results

Section S2: Cross-species comprehensive biomarker profile with top 35 biomarkers

Table S1: Top 30 biomarkers expression levels, function, and carcinogenicity potential

Figure S2: Stouffer integration biomarkers expression

Figure S3: SERPINE1 gene expression levels and analysis

Figure S4: FN1 gene expression levels and analysis

Figure S5: ID1 gene expression levels and analysis

Figure S6: ALDOA gene expression levels and analysis

Figure S7: PLIN2 gene expression levels and analysis

Figure S8: TRIB3 gene expression levels and analysis

Figure S9: ALDH3A2 gene expression levels and analysis

Figure S10: TSC22D3 gene expression levels and analysis

Section 3: PFOS/PFOA Exposure Alters Upstream Regulators

Section S1: Batch Effect Assessment and Meta-Analysis Validation

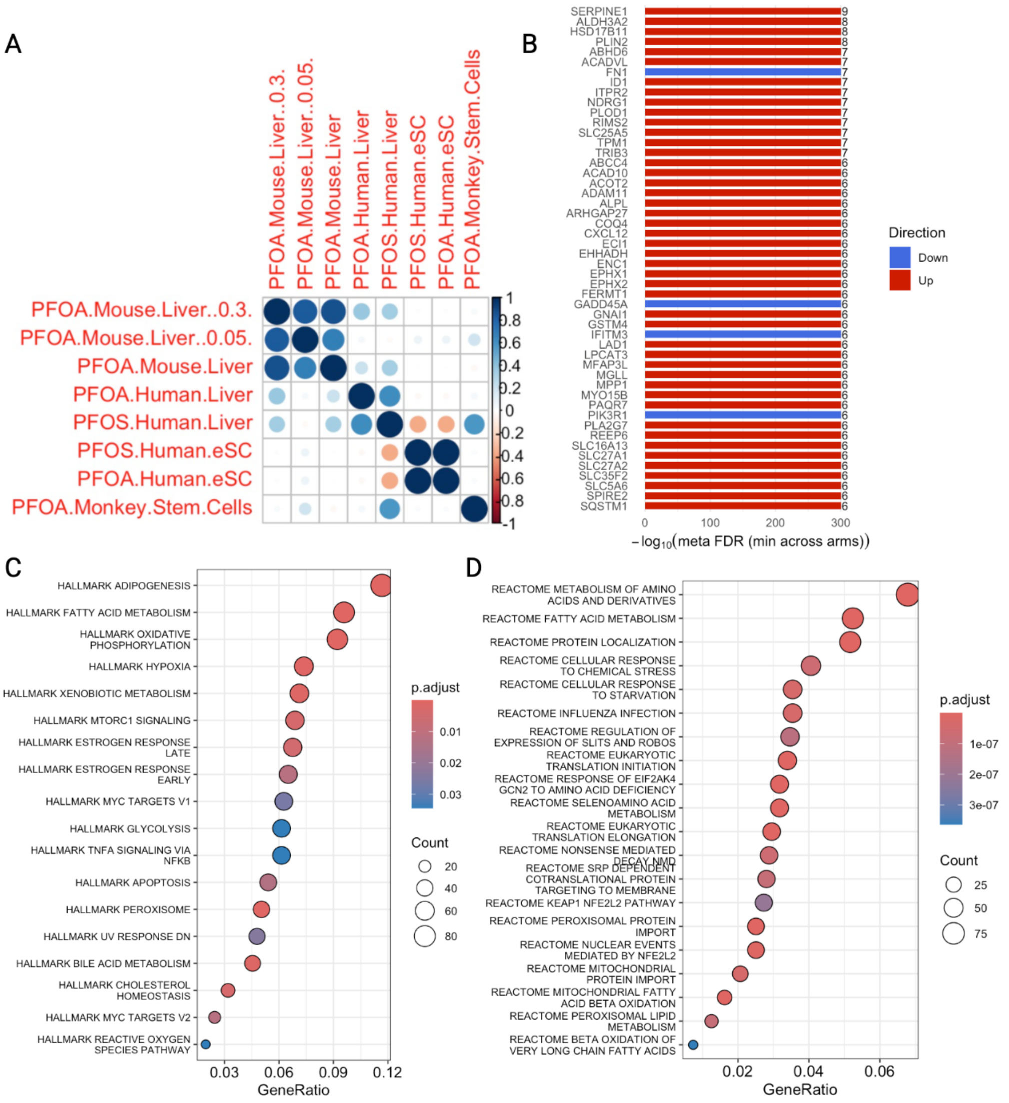

Figure S1: (A) Correlation plot of all datasets exposed to either PFOS or PFOA analyzed in this study, the color gradient ranges from blue (denoting negative correlation) to red (denoting positive correlation) (B) Top 50 overlapping genes between individual and meta analysis. The bar length correlates to the meta significance, the number at the right end indicates the number of DeSeq2 datasets the gene is significant in, and the color is a meta consensus direction prediction (upregulated is red, downregulated is blue).(C) Dotplot of pathway enrichment performed with the Hallmark gene set (D) Dotplot of pathway enrichment performed with the Reactome gene set.

In order to evaluate the differential responses of PFOS and PFOA across various species and tissue types across our samples, we used the cor() function in R and calculated the pairwise Pearson correlation coefficients between datasets. The corresponding correlation matrix generated is presented in Figure S8A. Our analysis revealed strong correlations between PFOS and PFOA exposures within the same species and tissue types, as well as among

interspecies samples with the same tissue types Figure S8A. The mouse liver samples demonstrated strong positive correlations among itself, and positive correlations with the human liver samples as well. The embryonic stem cell samples revealed strong positive correlations within human samples, and slightly positive across species. In all, the correlation plot matrix results support within species and inter species conserved transcriptional responses.

To further confirm our study approach, we performed a p-value combination meta-analysis using Stouffer's weighted Z and Fisher's methods across human, mouse, and monkey datasets. Results of the meta-analysis were consistent with the independent DESeq2 findings, supporting the reliability of the conserved responses. A robust DESeq2 gene set (genes significant in ≥4 datasets) was compared with the meta-analysis significant gene set to determine overlapping genes. The top 50 overlapping genes were retained, ranked by minimum meta-analysis FDR, and visualized as barplots with bar length reflecting $-\log_{10}$(min FDR), bar color denoting consensus direction (up- or down-regulation), and annotation showing the number of DESeq2 datasets in which each gene was significant (Figure S1B). These top biomarkers represent genes consistently dysregulated across multiple datasets and reinforced by both individual and meta-analysis approaches, providing high confidence in their relevance. Furthermore, a pathway enrichment of the meta-analysis revealed signatures of reactive oxygen species signaling, lipid metabolism, MYC signaling, amino acid metabolism, and protein translation were disrupted (Figure S1C & S1D). These pathways mirror the results of the individual DESeq2 analyses, demonstrating that the meta-analysis recovered similar biological processes. All together, these results confirm that the individual dataset analyses are accurate and robust, while the metaRNASeq approach strengthens confidence in the conserved biomarkers and pathways driving PFOS and PFOA carcinogenic responses.

Section S2: Cross-Species PFOS/PFOA Induced Carcinogenicity Biomarker Profile

Table S1: 35 top biomarkers identified in this study comprising the comprehensive biomarker profile with their cancer associations, function, classification, and cancer associations.

Gene	Up/Down Regulation	Cancer Association based on expression levels	Biomarker Classification	Cancers Associated
LAMP3	Upregulated	Regulates autophagy and EMT, facilitating tumor cell survival and metastasis [1-3]	Prognostic	Gastrointestinal, Cervical, Breast, and Esophageal squamous cell carcinoma[2]
TRIB3	Upregulated	Modulates stress signaling and immune evasion, allowing tumor cells to persist and grow.[4] [5,6]	Prognostic	Breast cancer, Liver cancer, Lung cancer, Glioblastomas, Ovarian cancer, Oral squamous cell carcinoma, Gastric cancer, Colorectal cancer, Bladder cancer[6], and Renal cell carcinoma[5]
SERPINE1	Upregulated	Controls fibrinolysis and angiogenesis, supporting tumor vascularization and invasion [7-9]	Therapeutic, Prognostic	Breast cancer, Colon adenocarcinoma, Stomach adenocarcinoma, Glioblastomas, Kidney renal clear cell carcinoma, and Head and neck squamous cell carcinoma[8]
HSD17B11	Upregulated	Alters steroid hormone metabolism, promoting proliferation and tumor progression [11] [12]	Prognostic	Colorectal cancer[12]
NUCB2	Downregulated	Influences cell adhesion and apoptosis, enabling cancer cells to evade death and become invasive.[13] [14,15]	Prognostic	Gastric cancer[16]
LDLR	Downregulated	Mediates cholesterol uptake, fueling lipid metabolism to support tumor cell growth. [17,18]	Prognostic	Hepatocellular carcinoma, Prostate cancer, and Cervical cancer[17]
FOXO3	Downregulated	Regulates transcription of tumor suppressors and apoptosis, its loss drives unchecked proliferation [19,20] [21]	Prognostic, Diagnostic, potential therapeutic target	Breast cancer, Ovarian cancer, Colorectal cancer[22],Gastric Cancer[21], and Prostate cancer[23]
SDC1	Downregulated	Maintains ECM structure; its decrease promotes cell migration and cancer dissemination. [24,25]	Prognostic, Diagnostic	Hepatocellular carcinoma, Small cell lung cancer, Colorectal cancer, and Prostate cancer[24]
ACTA1	Downregulated	Supports cytoskeletal integrity; its downregulation enhances cancer cell motility and invasion. [26] [27]	Prognostic	Head and neck squamous cell carcinoma, Colorectal cancer, Prostate cancer, and Pancreatic adenocarcinoma[26]
TSC22D3	Downregulated	Controls inflammation and immune response; its suppression fosters a tumor-promoting environment.	Prognostic	Acute myeloid leukemia[28]

		[28,29]		
FN1	Downregulated	Mediates ECM adhesion; its loss destabilizes tissue and enhances cancer cell dissemination [30] [31]	Prognostic	Colorectal cancer[32], Melanoma[33], and Ovarian cancer[34]
CYP3A5	Downregulated	Participates in metabolism of xenobiotics and steroids; upregulation increases ROS and DNA damage [35]	Prognostic	Hepatocellular carcinoma, Gastric cancer, Cervical cancer, Adrenal gland cortical carcinoma, Biliary Tract Cancers[36], Breast cancer[37], and Glioblastomas[38]
GADD45A	Upregulated	Involved in DNA repair under stress; its upregulation promotes adaptation and genomic instability [40].	Prognostic	Breast cancer, Pancreatic carcinoma[41]
ID1	Upregulated	Promotes cancer progression by suppressing dendritic cell differentiation and T-cell proliferation, facilitating immune evasion.[42], [43]	Prognostic	Lung cancer, Acute myeloid leukemia, Breast cancer, Prostate cancer, Colorectal cancer, Hepatocellular carcinoma, Glioblastomas, Thyroid cancer, and Cervical cancer[42]
ALDOA	Upregulated	Enhances cytoskeletal remodeling and supports EMT, aiding tumor invasion and metastasis [44] [45]	Diagnostic/Prognostic	Lung adenocarcinoma, Clear cell renal cell carcinoma, Gastric cancer, Pancreatic cancer[46], Hepatocellular carcinoma, Colorectal cancer, Cervical cancer, and Retinoblastoma[47]
PSAT1	Upregulated	Accelerates proliferation and cell cycle progression, which sustains rapid tumor growth and tumorigenesis [48]	Prognostic	Lung adenocarcinoma, breast invasive carcinoma, Kidney cancers[48], Clear cell renal cell carcinoma, ovarian cancer[49], and Endometrial carcinoma[50]
PLIN2	Upregulated	Promotes lipid accumulation, creating a pro-metastatic microenvironment that fuels cancer cell survival and spread [51] [52]	Diagnostic/Prognostic	Burkitt lymphoma, Colorectal cancer, Liver cancer, Melanoma, Gastric carcinoma[52], Clear cell renal cell carcinoma[51], and Oral squamous cell carcinoma[53]
CDH2	Downregulated	Its downregulation disrupts cell-cell contacts and tissue integrity, increasing tumor cell motility and invasive potential. [54] [55]	Diagnosis/Prognosis	Adrenocortical carcinoma[55]
EIF4EBP1	Upregulated	The upregulation drives protein synthesis and proliferation, facilitating uncontrolled tumor cell growth. [56] [57]	Prognostic	Breast cancer[57], Liver cancer, Neuroblastoma, Gliomas[58]
ERBB3	Upregulated	Increased proliferation and co-activation of IL-8	Predictive	Breast cancer, Ovarian cancer, Lung cancer, Colorectal cancer,

		signaling, which supports tumor invasiveness and metastatic progression..[59]		Melanoma, Cervical cancer, Prostate cancer, and Renal cell carcinoma[59]
GLUL	Downregulated	Downregulation reduces glutamine availability, forcing cancer cells to reprogram metabolism in ways that enhance their growth and survival. [60] [61]	Prognostic	Gastric cancer, Gliomas, and Liver cancer[61,62]
NQO1	Upregulated	Strengthens cancer cells' defenses against oxidative stress, allowing continued proliferation despite DNA-damaging conditions. [63]	Prognostic	Breast cancer, Pancreatic cancer, Colorectal cancer, Cholangiocarcinoma, Cervical cancer, Melanoma, Lung cancer[64], Glioma, Head and neck squamous cell carcinoma, Kidney renal papillary cell carcinoma, and Adrenocortical carcinoma[63]
COMT	Downregulated	Increases cellular stress and proliferation signaling which encourages tumor cell survival and growth. [65] [6]	Prognostic	Prostate cancer[66] and Breast cancer[65]
HSPD1	Upregulated	Supports cancer cell survival and adaptation under metabolic and inflammatory stress. [67] [68]	Prognostic	Oral squamous cell carcinoma[68,69], Gastric cancer[70], Large bowel carcinoma[71], Pancreatic cancer[72], Hepatocellular carcinoma[73]
RPL9	Upregulated	Increases translational capacity that can support tumor cell proliferation and a pro-survival state.[74]	Prognostic	Colorectal carcinoma, Small cell lung carcinoma, and Lung adenocarcinoma[74,75]
RPS6	Upregulated	increases protein synthesis and promotes proliferation and cell cycle progression. [76]	Prognostic	Ovarian cancer[77], Pancreatic cancer[78], Gastric cancer[76,79], Lung cancer, Renal cell carcinoma[80], and Hepatocellular carcinoma[81].
INSIG1	Downregulated	Disrupts lipid and glucose homeostasis, allowing cancer cells to adopt a more aggressive, metabolically adaptable phenotype.[82] [83,84]	Prognostic	Breast cancer and Prostate cancer[83,84]
PTGFR	Upregulated	Its upregulation enhances tumor-driven angiogenesis, supporting cancer cell growth and metastasis. [85]	Prognostic	Renal cell carcinoma[85]
NDRG1	Upregulated	Its upregulation induces mesenchymal-like changes and angiogenesis, driving cancer cell proliferation and aggressive behavior. [86] [87,88]	Prognostic	Lung cancer, Cervical cancer, Gastric cancer, Breast cancer, Hepatocellular carcinoma, Neuroblastoma, Thyroid carcinoma, Gallbladder carcinoma, and Esophageal squamous cell carcinoma[89]
PLCB2	Upregulated	Its upregulation enhances signal transduction supporting mitotic activity, EMT, and invasive tumor progression [90] [1,92]	Diagnostic/Prognostic	Breast cancer, Renal cell carcinoma, and Melanoma[90-92]

Figure S2

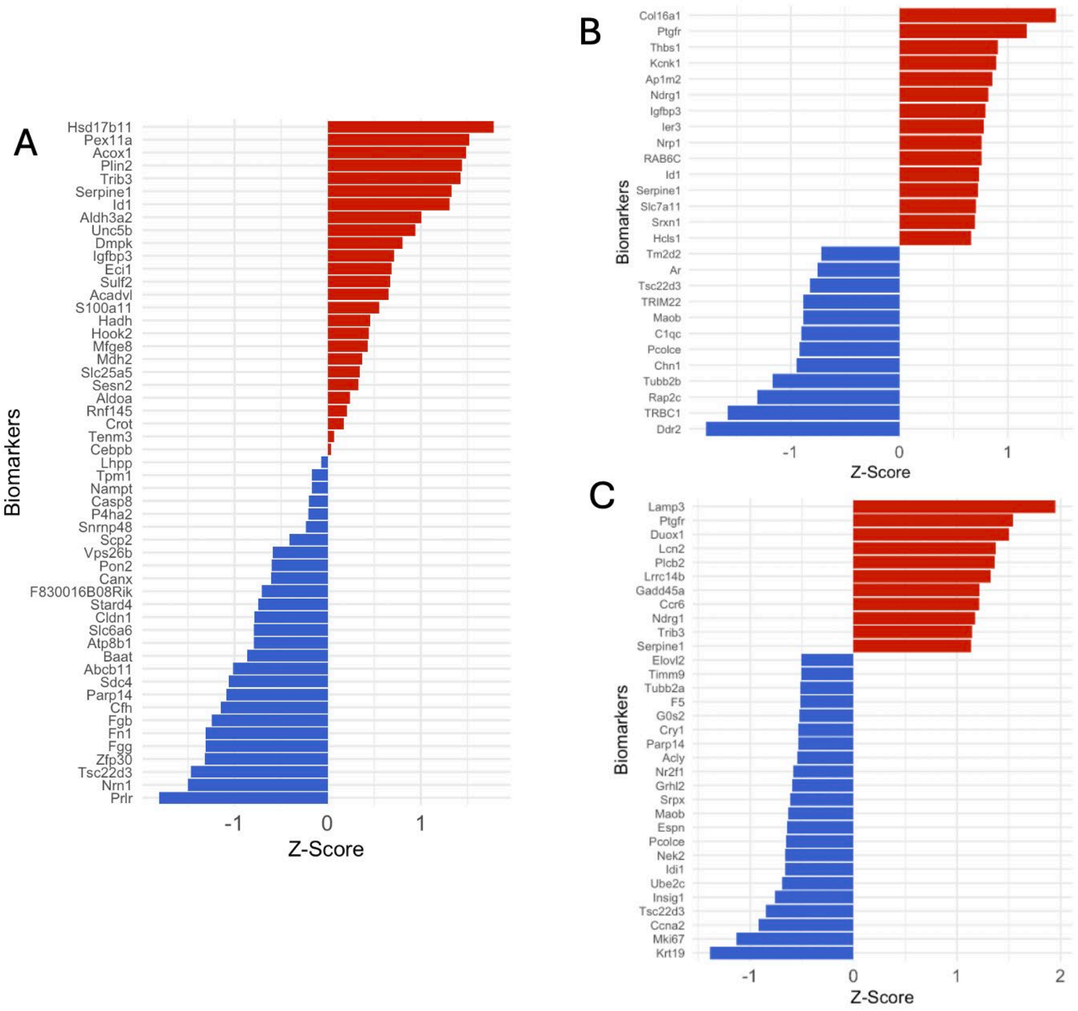

Figure S2: The regulation of biomarkers from the integrated gene expression analysis. Each biomarker's z-score was calculated using SD and Stouffer coefficients in RStudio to normalize expression.Biomarkers were filtered by |z-score| > 2 Biomarkers with positive z-scores (red) indicate upregulated genes, while negative z-scores (blue) indicate downregulated genes. **(A)** The top 25 biomarkers for PFOA exposure, identified in at least three out of four integrated tissue types. (**B)** The top biomarkers for PFOS exposure across all analyzed tissue types. **(C)** The top biomarkers present in all PFOS/PFOA exposed tissue types.

Figure S3

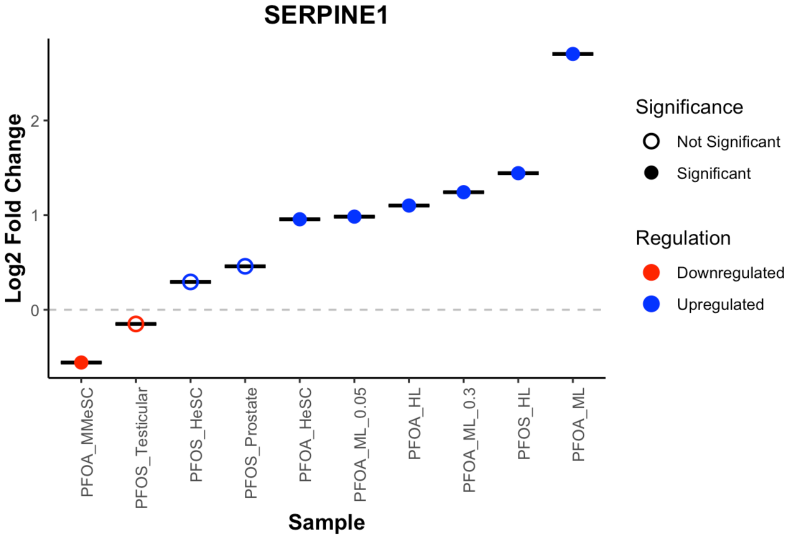

Figure S3: Gene expression data of Serpine1. The fold change, up (>0) or down regulation (<0) of the gene is indicated on the y axis. The x-axis indicated the sample information of the dataset. Red indicates a significant p value, <0.05, blue indicates a p value >0.05, and the gray indicates no value provided or calculated (NA

Serine Protease Inhibitor family E member 1 (SERPINE1) or plasminogen activator inhibitor-1 (PAI-1) has been recognized as a reliable prognostic biomarker for various cancers due to its key roles in the development and progression of cancer.[10] Studies have repeatedly revealed that SERPINE1 is upregulated in a majority of cancers and is associated with worse overall survival (OS) and disease-free survival.[10,93–99] Our analysis of DEGs found that SERPINE1 was the single most frequently upregulated gene in samples exposed to PFOS and PFOA, suggesting that SERPINE1 may be a key player in the tumor promoting mechanism of PFAS molecules. Supporting our finding, a recent study also discovered that PFOA exposure induced a marked increase in SERPINE1 levels.[100]

SERPINE1 is implicated in tumor progression via a variety of mechanisms, including remodeling of the tumor microenvironment (TME), angiogenesis, cancer cell migration, and inhibition of apoptosis.[9,94] SERPINE1 expression promotes tumor and immune related pathway activation which suggests that it may regulate the tumor immune microenvironment to aid immune cell infiltration and cancer progression.[9] Moreover, SERPINE1 overexpression has been associated with increased expression of vascular endothelial growth factor (VEGF) and IL-6, which are known to promote tumor angiogenesis and uncontrolled immune responses.[10] Other studies have demonstrated that SERPINE1 expression inhibits the spontaneous apoptosis of cancer cells, as well as catalyze the degradation of the basement membrane and ECM of normal cells.[93,101] Hence, SERPINE1 promotes pro-tumorigenic effects through several

mechanisms which proves a significant role in cancer development and progression. Given this, our finding that PFOS and PFOA exposure upregulates SERPINE1 expression may indicate a higher risk for cancer development.

Figure S4

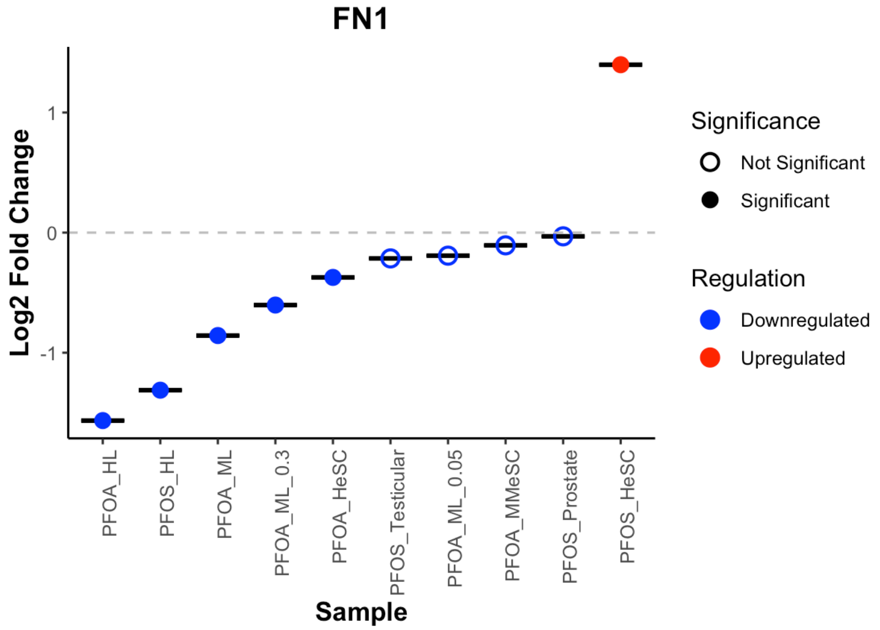

Figure S4: Gene expression data of Fn1. The fold change, up (>0) or down regulation (<0) of the gene is indicated on the y axis. The x-axis indicated the sample information of the dataset. Red indicates a significant p value, <0.05, blue indicates a p value >0.05, and the gray indicates no value provided or calculated (NA).

Fibronectin 1 (FN1) is an extracellular matrix (ECM) glycoprotein that is known to play a role in cancer onset and progression. Specifically, most studies have shown that FN1 is overexpressed in various cancers and facilitates cancer cell proliferation and survival.[102–106] This is explained by the role of FN1 as a matrix component in the tumor niche that generates an ideal tumor microenvironment (TME) which sustains cancer cell survival, migration, and angiogenesis.[107] For instance, FN1 can bind directly to and regulate VEGF, which is known to induce tumor angiogenesis.[107]

Interestingly, our analysis found that FN1 was significantly downregulated in the majority of PFOA/PFOS-exposed samples and upregulated in only one sample. This finding may be explained by recent studies reporting that FN1 can also play an opposite role of inhibiting cell invasion and cancer metastasis in certain cancers and contexts.[108] For instance, downregulated FN1 in sporadic medullary thyroid cancer tissues was an independent predictor of poor prognosis[109]. Additionally, polypeptides derived from FN1 inhibited adhesion and invasion of liver cancer cells in another study.[110] Similarly, a recent study found that FN1 can inhibit cancer metastasis and that neuroblastoma patients with lower levels of FN1 had worse OS.[108] These inconsistent findings may point to an understanding that dysregulated FN1 expression in general can confer pro-tumorigenic effects, with upregulation and downregulation of the gene causing different carcinogenic effects in different contexts. Hence, the downregulation and, to a

lesser extent, upregulation of FN1 by PFOA/PFOS exposure in our analysis may indicate an overall dysregulated expression and increased risk of cancer onset and progression, given previous findings of the FN1 gene's dual role in cancer.

Figure S5

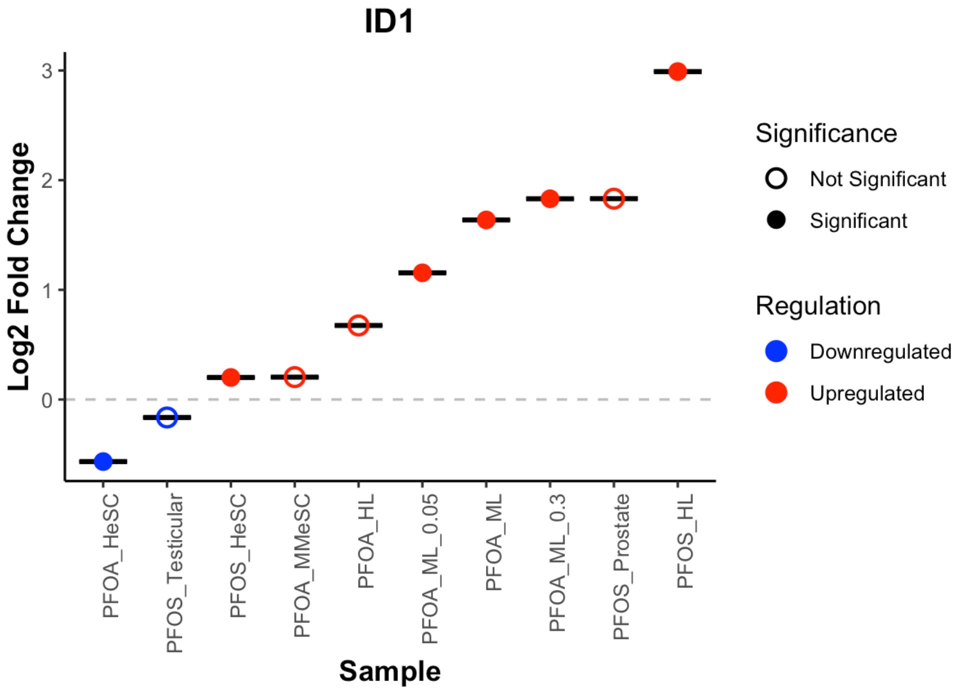

Figure S5: Gene expression data of Id1. The fold change, up (>0) or down regulation (<0) of the gene is indicated on the y axis. The x-axis indicated the sample information of the dataset. Red indicates a significant p value, <0.05, blue indicates a p value >0.05, and the gray indicates no value provided or calculated (NA).

Across our analysis of DEGs, the inhibitor of differentiation 1 (ID1) gene was upregulated in the majority of samples with significant gene expressions. ID proteins are key regulators of the cell cycle and cell differentiation, among which ID1 has been recognized as most closely linked to tumorigenesis.[42] ID1 is upregulated in numerous cancer types and is often considered a tumor promoter.[42] Moreover, the upregulation of ID1 has been correlated with cancer progression and poor prognosis.[43] Notably, upregulated ID1 plays an important role in tumor angiogenesis, a process largely triggered by the upregulation of VEGF.[42] Studies have shown that TGF-β1 in ID1 pathways regulate VEGF expression, meaning upregulation of ID1 can lead to higher expression of VEGF, which in turn triggers tumor angiogenesis.[42] Hence, the upregulation of ID1 by PFOS and PFOA exposure in our samples suggests an increased risk for tumorigenesis and tumor progression via tumor angiogenesis, which sustains sufficient nutrients for cancer cell proliferation.

Another central mechanism of tumor progression and metastasis is immunosuppression, which inhibits effective anti-tumor responses by the immune system.[43] Tumors achieve this by preventing the differentiation of myeloid cells into functional antigen-presenting cells (APCs), such as dendritic cells (DCs).[43] The resulting immature myeloid cells are known as myeloid-derived suppressor cells (MDSCs). Recent studies have found that the upregulation of ID1 promotes this dysfunctional differentiation of myeloid cells into MDSCs, with an associated decrease in DCs.[43] The decline in DCs inevitably leads to an inefficient immune response, while

the increase in MDSCs has been shown to suppress CD8 T-cell function and proliferation.[43] Hence, by upregulating the ID1 gene, PFOS and PFOA exposure is implicated in generating an ideal immunosuppressive environment for tumors, increasing the risk for primary tumor growth and metastatic progression.

Figure S6

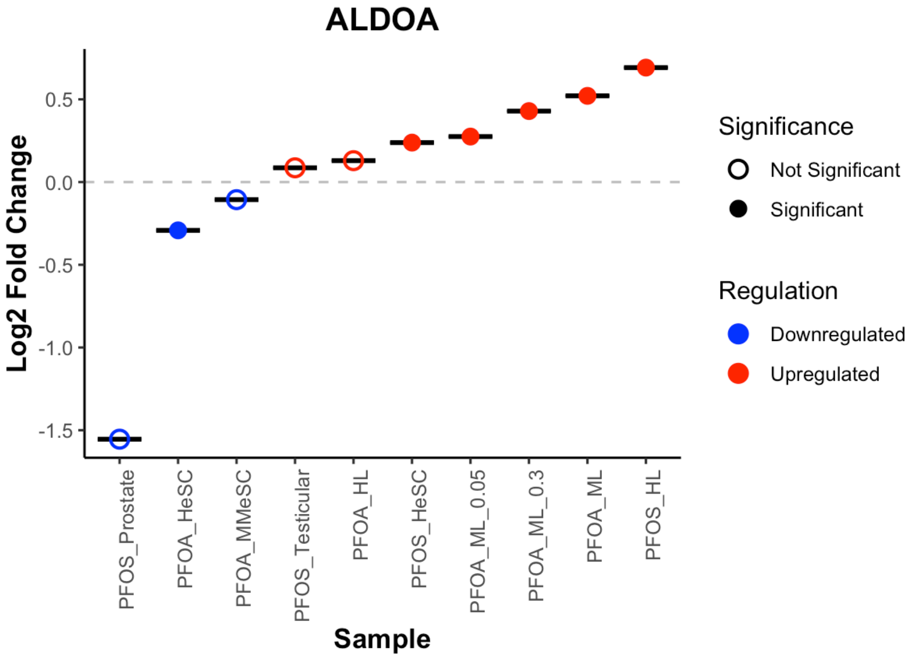

Figure S6: Gene expression data of Aldoa. The fold change, up (>0) or down regulation (<0) of the gene is indicated on the y axis. The x-axis indicated the sample information of the dataset. Red indicates a significant p value, <0.05, blue indicates a p value >0.05, and the gray indicates no value provided or calculated (NA).

Metabolic reprogramming is a hallmark of cancer cells, which require an increased energy supply to sustain their uncontrolled growth and proliferation. In particular, glucose metabolism dysfunction is one of the most common metabolic rewirings in cancer, as glycolysis is the main source of energy for the survival of cancer cells.[46] Aldolase is a key enzyme family in glucose metabolism, with aldolase A (ALDOA) being the most abundant isoform in cancers.[46] Our analysis of DEGs revealed that the ALDOA gene was significantly upregulated in the majority of samples exposed to PFOS/PFOA. This finding is in agreement with previous studies that have demonstrated upregulation of ALDOA in several tumors, associated with the initiation and progression of cancer as well as OS and prognosis.[45,111] As a critical enzyme in glucose metabolism pathways, ALDOA can promote cancer development and metastasis by accelerating glycolysis.[112]

In addition to this central function, in one study, ALDOA was found to interact with IGF2BP1 to facilitate its binding to eIF4G mRNA This can promote eIF4G protein synthesis and thereby increase oncogenic translation in hepatocellular carcinoma (HCC).[112] Another study found that ALDOA overexpression ensures tumor cells maintain their actin cytoskeleton integrity during the epithelial–mesenchymal transition which contributes to cancer cell survival and proliferation.[45] Owing to these pro-tumorigenic functions of ALDOA, several studies have proposed ALDOA as an oncogene and promising biomarker for certain cancers, such as HCC.[46,111,113,114] Hence, our finding that PFOS/PFOA exposure upregulates the ALDOA gene likely indicates a greater risk for cancer development.

Figure S7

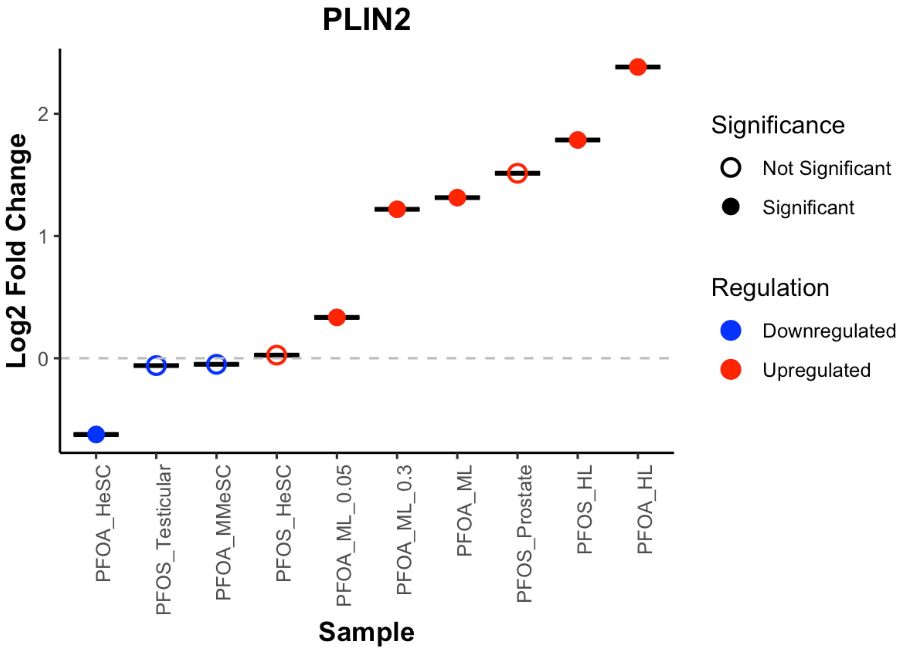

Figure S7: Gene expression data of Plin2. The fold change, up (>0) or down regulation (<0) of the gene is indicated on the y axis. The x-axis indicated the sample information of the dataset. Red indicates a significant p value, <0.05, blue indicates a p value >0.05, and the gray indicates no value provided or calculated (NA).

Our study found that the adipophilin/perilipin-2 (PLIN2) gene was significantly upregulated in most samples exposed to PFOS/PFOA, which suggests a role for this gene in facilitating the pro-tumerogenic effects of PFAS exposure. Consistently, numerous studies have found that PLIN2 is overexpressed in several cancers.[51,115–118] Tumor cells synthesize and store lipids in the form of lipid droplets (LDs) as critical energy reservoirs for coping with insufficient nutrient supplies during their rapid proliferation.[119] PLIN2 exists in LDs in cells, serving as a key protein involved in LD synthesis, an important process for cancer cell survival.[119] Specifically, it has been reported that downregulation of PLIN2 degrades the HIF1α protein, an important messenger that activates the transcriptional response to hypoxia.[119] Tumors are characterized by hypoxia, meaning decreased levels of HIF1α prohibits cancer cells from surviving hypoxic conditions. Hence, the upregulation of PLIN2 by PFOS/PFOA exposure may support cancer development by accelerating the production of the HIF1α protein and facilitating tumor cell survival against hypoxia.

Figure S8

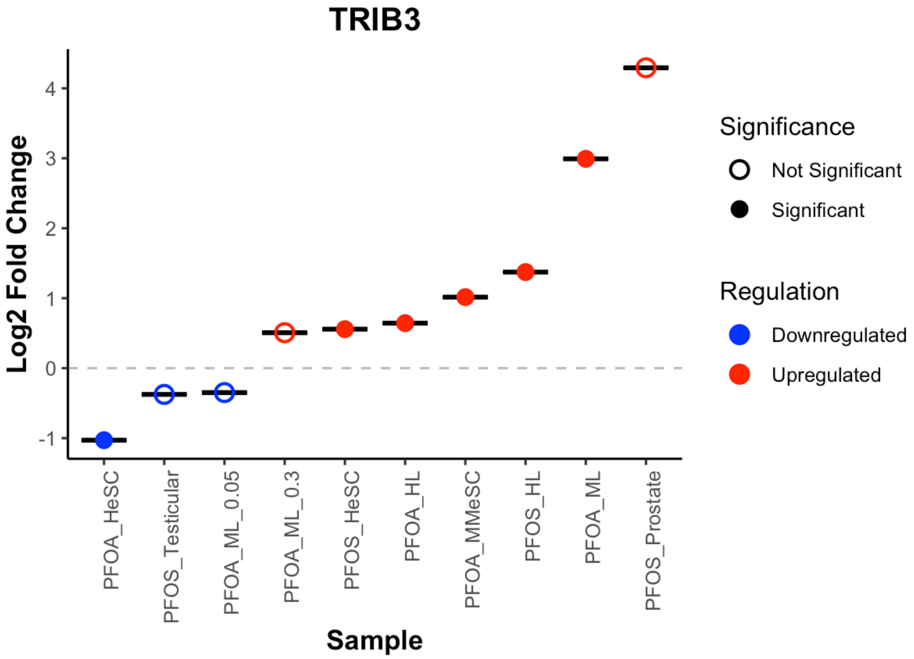

Figure S8: Gene expression data of Fn1. The fold change, up (>0) or down regulation (<0) of the gene is indicated on the y axis. The x-axis indicated the sample information of the dataset. Red indicates a significant p value, <0.05, blue indicates a p value >0.05, and the gray indicates no value provided or calculated (NA).

Among our analysis, the tribbles pseudokinase (TRIB3) gene was significantly upregulated in 5 samples and downregulated in 1 sample. TRIB3 has been largely studied and was found upregulated in several cancers where its role as an oncogene promotes tumor progression. TRIB3 is a stress-response protein, regulating signaling pathways in connection to cell survival, apoptosis, and proliferation.[120] As an oncogene, this suggests that the upregulation of TRIB3 promotes cancer, which was seen where it enhanced tumor cell survival with resistance toward stress.[6] A recent study saw that the overexpression of TRIB3 observed in RCC and lung adenocarcinoma patients correlated with poor prognosis.[5,121] It found molecular interactions between lipid-droplet associated protein PLIN2, previously discussed, and TRIB3, facilitating the accumulation of lipids and accelerating RCC tumor progression.[5] Furthermore, TRIB3 was recently reported to promote progression through its interaction with autophagic receptor p62, impairing the degradation functions of autophagy.[122] Furthermore, the dysregulation of TRIB3 in numerous studies suggests that it plays a role in cancer development, progression, and metastasis.[3] Therefore, it is likely that upregulation caused due to exposure by PFOS/PFOA may promote cancer development due to its interaction with PLIN2 and p62 and enhancement of cell survival.

Figure S9

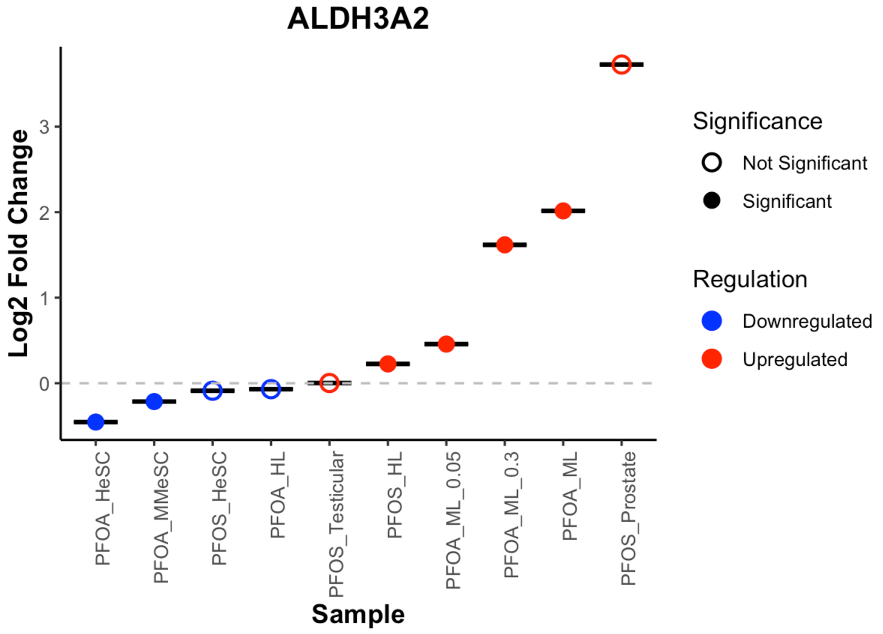

Figure S9: Gene expression data of Aldh3a2. The fold change, up (>0) or down regulation (<0) of the gene is indicated on the y axis. The x-axis indicated the sample information of the dataset. Red indicates a significant p value, <0.05, blue indicates a p value >0.05, and the gray indicates no value provided or calculated (NA

In our analysis of DEGs, the ALDH3A2 gene, a member of the aldehyde dehydrogenase family, was found to be significantly upregulated in 4 samples and downregulated in 2 samples. While our study found this gene mostly upregulated among PFAS-exposed samples upregulated, literature has shown that the lower expression levels of ALDH3A2 is prevalent among several cancers.[123–125] ALDH3A2 plays a critical role in the detoxification of aldehydes generated by metabolism and lipid peroxidation, in which downregulation may increase cellular vulnerability.[126] A recent study demonstrated that ALDH3A2 was downregulated or silenced in ovarian cancer, finding that high expression leads to poor prognosis. It suggested that ALDH3A2 regulates ferroptosis in tumors where the lower expression levels increase the lethal effects in ovarian cancer cells.[125] Furthermore, another study found that miR-1182 mediates the downregulation of ALDH3A2 in ccRCC, promoting tumor progression.[124] Functionally, ALDH3A2 exhibits tumor-suppressive properties that impact phenotypes of ccRCC cells, contributing to easier tumor progression. Thus, the downregulation of ALDH3A2 by PFOS/PFOA exposure may contribute to cancer development by the more vulnerability of the cells and tumor progression. Interestingly, the role of ALDH3A2 when upregulated has been largely undetermined in literature, thus additional research is needed before making conclusions in connection with PFOS/PFOA.

Figure S10

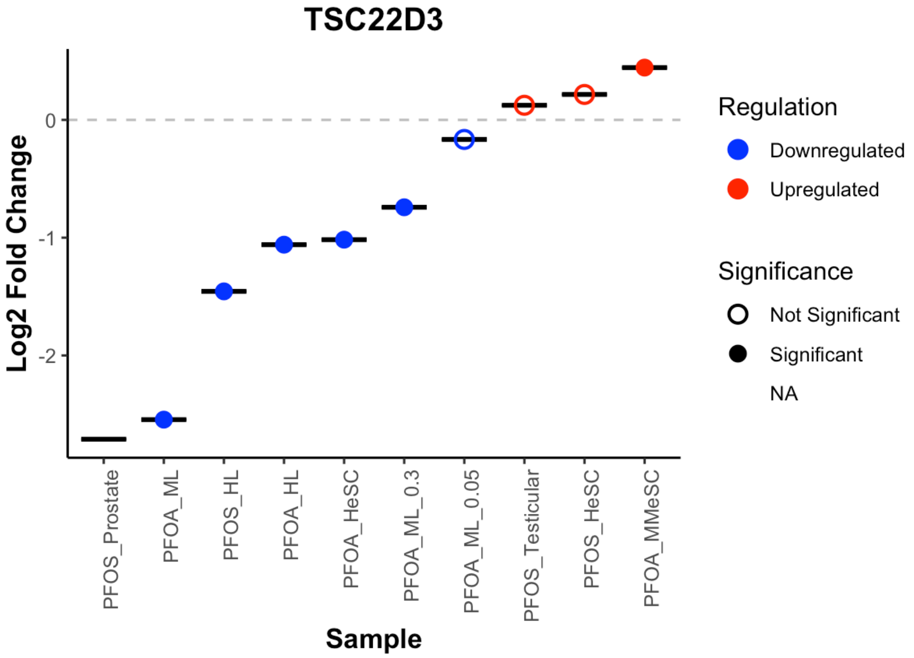

Figure S10: Gene expression data of Tsc22d3. The fold change, up (>0) or down regulation (<0) of the gene is indicated on the y axis. The x-axis indicated the sample information of the dataset. Red indicates a significant p value, <0.05, blue indicates a p value >0.05, and the gray indicates no value provided or calculated (NA).

Our study found that the Glucocorticoid gene TSC22D3 was significantly downregulated in most of our PFOS/PFOA-exposed samples. Currently, lower expression levels of TSC22D3 with cancer development have been closely studied, especially in association with acute myeloid leukemia (AML).[28] The role of TSC22D3 has been emphasized with its involvement in regulating immune responses, in which its downregulation contributes to tumorigenesis, immune evasion, and poor prognosis.[28] In a recent study, it indicated that the suppression of TSC22D3 can shift towards an immunosuppressive microenvironment with enhanced polarization of M2 macrophages, known to promote tumor evasion and progression of tumor cells.[127] Furthermore, its downregulation impairs the ability of macrophages. Specifically, by inhibiting NF-kB/NLRP3 signaling, TSC22D3 normally would help control tumor growth by producing pro-inflammatory cytokines, IL-1β. However, the lower expression of TSC22D3 has reduced release of the cytokines, inhibiting macrophage polarization of the M1 subtype, attenuating the tumor microenvironment.[28] Hence, the downregulation of TSC22D3 by PFOS/PFOA exposure may contribute to cancer development by the creation of a tumor microenvironment with reduced immune regulation.

Section 3: PFOS/PFOA Exposure Alters Upstream Regulators

The following upstream regulators were identified as significant; they have been analyzed by their function, expression levels, and their role in the key characteristics of carcinogens. SLC27A2 is a fatty acid transporter and its upregulation is associated with increased lipid uptake which supplies lipids for cancer cell membrane synthesis. It is associated with the upregulated pathways of fatty acid metabolism (Section 3.2.1). XBP1 is a key regulator of unfolded protein response and when overexpressed helps cancer cells adapt to stress. This regulator is associated with the highly upregulated pathway of electron transport, ATP synthesis, and heat production by coupling proteins which contributes to oxidative stress (Section 3.2.4). The adaptation of stress by cancer cells allows resistance to chemotherapy and increased risk of metastasis. PPARGC1A showed overexpression and controls mitochondrial function. The upregulation increases the ability of cancer cells to adapt to hypoxic conditions and is associated with the upregulated pathway of mitochondrial dysfunction (Section 3.2.4). The CLPP gene breaks down unwanted proteins in the mitochondria. It showed downregulation across all but one sample which demonstrates disruption in normal mitochondrial function that can lead to an abundance of unnecessary proteins. This downregulation is associated with several of the dysregulated protein synthesis pathways that contribute to alterations in cell proliferation, cell death, and nutrient supply (Section 3.2.2). In the liver samples, HNF1A, a transcription factor involved in liver specific gene regulation, was consistently downregulated. HNF1A is also a tumor suppressor, hence its underexpression disrupts liver cell proliferation and decreases apoptosis. Its downregulation is associated with immune suppression and the downregulated pathway of T-Cell receptor signaling (Section 3.2.3)

References

1. Wattanathavorn W, Seki M, Suzuki Y, Buranapraditkun S, Kitkumthorn N, Sasivimolrattana T, et al. Downregulation of LAMB3 Altered the Carcinogenic Properties of Human Papillomavirus 16-Positive Cervical Cancer Cells. Int J Mol Sci. 2024 Feb 22;25(5):2535.

2. Liao X, Chen Y, Liu D, Li F, Li X, Jia W. High Expression of LAMP3 Is a Novel Biomarker of Poor Prognosis in Patients with Esophageal Squamous Cell Carcinoma. Int J Mol Sci. 2015 Jul 31;16(8):17655–67.

3. Stefanovska B, André F, Fromigué O. Tribbles Pseudokinase 3 Regulation and Contribution to Cancer. Cancers. 2021 Apr 11;13(8):1822.

4. Wang RQ, He FZ, Meng Q, Lin WJ, Dong JM, Yang HK, et al. Tribbles pseudokinase 3 (TRIB3) contributes to the progression of hepatocellular carcinoma by activating the mitogen-activated protein kinase pathway. Ann Transl Med. 2021 Aug;9(15):1253–1253.

5. Li J, Zhang Q, Guan Y, Liao D, Chen H, Xiong H, et al. TRIB3 promotes the progression of renal cell carcinoma by upregulating the lipid droplet-associated protein PLIN2. Cell Death Dis. 2024 Apr 1;15(4):240.

6. Hu C, Li Q, Xiang L, Luo Y, Li S, An J, et al. Comprehensive pan-cancer analysis unveils the significant prognostic value and potential role in immune microenvironment modulation of TRIB3. Comput Struct Biotechnol J. 2024 Dec;23:234–50.

7. NCBI. SERPINE1 serpin family E member 1 [Homo sapiens (human)] - Gene [Internet]. National Institutes of Health (NIH). Available from: https://www.ncbi.nlm.nih.gov/gene/5054?report=full_report

8. Polo-Generelo S, Rodríguez-Mateo C, Torres B, Pintor-Tortolero J, Guerrero-Martínez JA, König J, et al. Serpine1 mRNA confers mesenchymal characteristics to the cell and promotes CD8+ T cells exclusion from colon adenocarcinomas. Cell Death Discov. 2024 Mar 6;10(1):116.

9. Wang S, Pang L, Liu Z, Meng X. SERPINE1 associated with remodeling of the tumor microenvironment in colon cancer progression: a novel therapeutic target. BMC Cancer. 2021 Dec;21(1):767.

10. Chen S, Li Y, Zhu Y, Fei J, Song L, Sun G, et al. SERPINE1 Overexpression Promotes Malignant Progression and Poor Prognosis of Gastric Cancer. J Oncol. 2022;2022:2647825.

11. NCBI. HSD17B11 hydroxysteroid 17-beta dehydrogenase 11 [Homo sapiens (human)] - Gene [Internet]. National Institutes of Health (NIH). Available from: https://www.ncbi.nlm.nih.gov/gene/51170

12. Zhang W, Wang B, Wang Q, Zhang Z, Shen Z, Ye Y, et al. Lnc-HSD17B11-1:1 Functions as a Competing Endogenous RNA to Promote Colorectal Cancer Progression by Sponging miR-338-3p to Upregulate MACC1. Front Genet. 2020 Jun 12;11:628.

13. Kan JY, Yen MC, Wang JY, Wu DC, Chiu YJ, Ho YW, et al. Nesfatin-1/Nucleobindin-2 enhances cell migration, invasion, and epithelial-mesenchymal transition via LKB1/AMPK/TORC1/ZEB1 pathways in colon cancer. Oncotarget. 2016 May 24;7(21):31336–49.

14. Xu H, Li W, Qi K, Zhou J, Gu M, Wang Z. A novel function of NUCB2 in promoting the development and invasion of renal cell carcinoma. Oncol Lett [Internet]. 2017 Dec 8 [cited 2025 Feb 8]; Available from: http://www.spandidos-publications.com/10.3892/ol.2017.7563

15. Ning S, Liu C, Wang K, Cai Y, Ning Z, Li M, et al. NUCB2/Nesfatin-1 drives breast cancer metastasis through the up-regulation of cholesterol synthesis via the mTORC1 pathway. J Transl Med. 2023 Jun 5;21(1):362.

16. Kalnina Z, Silina K, Bruvere R, Gabruseva N, Stengrevics A, Barnikol-Watanabe S, et al. Molecular characterisation and expression analysis of SEREX-defined antigen NUCB2 in gastric epithelium, gastritis and gastric cancer. Eur J Histochem EJH. 2009 Mar 31;53(1):e2.

17. Chen Z, Chen L, Sun B, Liu D, He Y, Qi L, et al. LDLR inhibition promotes hepatocellular carcinoma proliferation and metastasis by elevating intracellular cholesterol synthesis through the MEK/ERK signaling pathway. Mol Metab. 2021 Sep;51:101230.

18. Liu L, Sun Y, An R, Cheng R, Li N, Zheng J. LDLR promotes autophagy-mediated cisplatin resistance in ovarian cancer associated with the PI3K / AKT / mTOR signaling pathway. Kaohsiung J Med Sci. 2023 Aug;39(8):779–88.

19. Liu H, Song Y, Qiu H, Liu Y, Luo K, Yi Y, et al. Downregulation of FOXO3a by DNMT1 promotes breast cancer stem cell properties and tumorigenesis. Cell Death Differ. 2020 Mar;27(3):966–83.

20. Mei W, Mei B, Chang J, Liu Y, Zhou Y, Zhu N, et al. Role and regulation of FOXO3a: new insights into breast cancer therapy. Front Pharmacol. 2024 Mar 4;15:1346745.

21. Tsuji T, Maeda Y, Kita K, Murakami K, Saya H, Takemura H, et al. FOXO3 is a latent tumor suppressor for FOXO3-positive and cytoplasmic-type gastric cancer cells. Oncogene. 2021 Apr 29;40(17):3072–86.

22. Farhan M, Silva M, Li S, Yan F, Fang J, Peng T, et al. The role of FOXOs and autophagy in cancer and metastasis-Implications in therapeutic development. Med Res Rev. 2020 Nov;40(6):2089–113.

23. Park SH, Chung YM, Ma J, Yang Q, Berek JS, Hu MCT. Pharmacological activation of FOXO3 suppresses triple-negative breast cancer *in vitro* and *in vivo*. Oncotarget. 2106 Jul 5;7(27):42110–25.

24. Liu Z, Jin H, Yang S, Cao H, Zhang Z, Wen B, et al. *SDC1* knockdown induces epithelial–mesenchymal transition and invasion of gallbladder cancer cells via the ERK/Snail pathway. J Int Med Res. 2020 Aug;48(8):0300060520947883.

25. Ishikawa T, Kramer RH. Sdc1 negatively modulates carcinoma cell motility and invasion. Exp Cell Res. 2010 Apr;316(6):951–65.

26. Suresh R, Diaz RJ. The remodelling of actin composition as a hallmark of cancer. Transl Oncol. 2021 Jun;14(6):101051.

27. Hu Q, Zhu L, Li Y, Zhou J, Xu J. ACTA1 is inhibited by PAX3-FOXO1 through RhoA-MKL1-SRF signaling pathway and impairs cell proliferation, migration and tumor growth in Alveolar Rhabdomyosarcoma. Cell Biosci. 2021 Dec;11(1):25.

28. Li Y, Huang H, Zhu Z, Chen S, Liang Y, Shu L. TSC22D3 as an immune-related prognostic biomarker for acute myeloid leukemia. iScience. 2023 Aug;26(8):107451.

29. Kanda A, Hirose I, Noda K, Murata M, Ishida S. Glucocorticoid-transactivated TSC22D3 attenuates hypoxia- and diabetes-induced Müller glial galectin-1 expression via HIF-1α destabilization. J Cell Mol Med. 2020 Apr;24(8):4589–99.

30. NCBI. FN1 fibronectin 1 [Homo sapiens (human)]- Gene [Internet]. National Institutes of Health (NIH). Available from: https://www.ncbi.nlm.nih.gov/gene/2335

31. Liu M, Chen P, Wei B, Tan HL, Zhao YX, Ai L, et al. FN1 shapes the behavior of papillary thyroid carcinoma through alternative splicing of EDB region. Sci Rep. 2025 Jan 2;15(1):327.

32. Cai X, Liu C, Zhang T, Zhu Y, Dong X, Xue P. Down-regulation of FN1 inhibits colorectal carcinogenesis by suppressing proliferation, migration, and invasion. J Cell Biochem. 2018 Jun;119(6):4717–28.

33. Li B, Shen W, Peng H, Li Y, Chen F, Zheng L, et al. Fibronectin 1 promotes melanoma proliferation and metastasis by inhibiting apoptosis and regulating EMT. OncoTargets Ther. 2019 May;Volume 12:3207–21.

34. Zhang H, Liu M, Zhong H, Ma L, Liu Y, Liu C, et al. Mechanistic role of FN1 in LAIR-1 mediated downregulation of ovarian cancer cell proliferation. BMC Cancer. 2025 Feb 25;25(1):339.

35. Shao M, Pan Q, Tan H, Wu J, Lee HW, Huber AD, et al. CYP3A5 unexpectedly regulates glucose metabolism through the AKT–TXNIP–GLUT1 axis in pancreatic cancer. Genes Dis. 2024 Jul;11(4):101079.

36. Noll EM, Eisen C, Stenzinger A, Espinet E, Muckenhuber A, Klein C, et al. CYP3A5 mediates basal and acquired therapy resistance in different subtypes of pancreatic ductal adenocarcinoma. Nat Med. 2016 Mar;22(3):278–87.

37. Lamba J, Hebert JM, Schuetz EG, Klein TE, Altman RB. PharmGKB summary: very important pharmacogene information for CYP3A5. Pharmacogenet Genomics. 2012 Jul;22(7):555–8.

38. Hu W, Cui X, Liu H, Li Z, Chen X, Wang Q, et al. CYP3A5 promotes glioblastoma stemness and chemoresistance through fine-tuning NAD+/NADH ratio. J Exp Clin Cancer Res. 2025 Jan 3;44(1):3.

39. NCBI. GADD45A growth arrest and DNA damage inducible alpha [Homo sapiens (human)]- Gene [Internet]. National Institutes of Health (NIH). Available from:

https://www.ncbi.nlm.nih.gov/gene/1647

40. Tront JS, Huang Y, Fornace AJ, Hoffman B, Liebermann DA. Gadd45a functions as a promoter or suppressor of breast cancer dependent on the oncogenic stress. Cancer Res. 2010 Dec 1;70(23):9671–81.

41. Palomer X, Salvador JM, Griñán-Ferré C, Barroso E, Pallàs M, Vázquez-Carrera M. GADD45A: With or without you. Med Res Rev. 2024 Jul;44(4):1375–403.

42. Zhao Z, Bo Z, Gong W, Guo Y. Inhibitor of Differentiation 1 (Id1) in Cancer and Cancer Therapy. Int J Med Sci. 2020;17(8):995–1005.

43. Papaspyridonos M, Matei I, Huang Y, Do Rosario Andre M, Brazier-Mitouart H, Waite JC, et al. Id1 suppresses anti-tumour immune responses and promotes tumour progression by impairing myeloid cell maturation. Nat Commun. 2015 Apr 29;6(1):6840.

44. Pirovich DB, Da'dara AA, Skelly PJ. Multifunctional Fructose 1,6-Bisphosphate Aldolase as a Therapeutic Target. Front Mol Biosci. 2021;8:719678.

45. Gizak A, Wiśniewski J, Heron P, Mamczur P, Sygusch J, Rakus D. Targeting a moonlighting function of aldolase induces apoptosis in cancer cells. Cell Death Dis. 2019 Sep 26;10(10):712.

46. Tang Y, Yang X, Feng K, Hu C, Li S. High expression of aldolase A is associated with tumor progression and poor prognosis in hepatocellular carcinoma. J Gastrointest Oncol. 2021 Feb;12(1):174–83.

47. Wang Y, Tang J, Liu Y, Zhang Z, Zhang H, Ma Y, et al. Targeting ALDOA to modulate tumorigenesis and energy metabolism in retinoblastoma. iScience. 2024 Sep;27(9):110725.

48. Feng M, Cui H, Tu W, Li L, Gao Y, Chen L, et al. An integrated pan-cancer analysis of PSAT1: A potential biomarker for survival and immunotherapy. Front Genet. 2022 Aug 29;13:975381.

49. Zhu S, Wang X, Liu L, Ren G. Stabilization of Notch1 and β-catenin in response to ER- breast cancer-specific up-regulation of PSAT1 mediates distant metastasis. Transl Oncol. 2022 Jun;20:101399.

50. Wang M, Yue S, Yang Z. Downregulation of PSAT1 inhibits cell proliferation and migration in uterine corpus endometrial carcinoma. Sci Rep. 2023 Mar 11;13(1):4081.

51. Cao Q, Ruan H, Wang K, Song Z, Bao L, Xu T, et al. Overexpression of PLIN2 is a prognostic marker and attenuates tumor progression in clear cell renal cell carcinoma. Int J Oncol [Internet]. 2018 Apr 26 [cited 2025 Feb 8]; Available from: http://www.spandidos-publications.com/10.3892/ijo.2018.4384

52. Li ML, Luo HY, Quan ZW, Huang LT, Wang JH. Prognostic and clinicopathologic significance of PLIN2 in cancers: A systematic review with meta-analysis. Int J Biol Markers. 2023 Mar;38(1):3–14.

53. Zhang J, Peng J, Wang S, Wang L, Sun Y, Xia J, et al. Perilipin2-dependent lipid droplets accumulation promotes metastasis of oral squamous cell carcinoma via epithelial-mesenchymal transition. Cell Death Discov. 2025 Jan 28;11(1):30.

54. Lu CH, Wu CH, Hsieh PF, Wu CY, Kuo W, Ou CH, et al. Small interfering RNA targeting N-cadherin regulates cell proliferation and migration in enzalutamide-resistant prostate cancer. Oncol Lett. 2022 Jan 21;23(3):90.

55. Situ Y, Deng L, Huang Z, Jiang X, Zhao L, Zhang J, et al. *CDH2* and *CDH13* as potential prognostic and therapeutic targets for adrenocortical carcinoma. Cancer Biol Ther. 2024 Dec 31;25(1):2428469.

56. Nelson ED. High EIF4EBP1 expression reflects mTOR pathway activity and cancer cell proliferation and is a biomarker for poor breast cancer prognosis. Am J Cancer Res. 2024;14(1):227–42.

57. Rutkovsky AC, Yeh ES, Guest ST, Findlay VJ, Muise-Helmericks RC, Armeson K, et al. Eukaryotic initiation factor 4E-binding protein as an oncogene in breast cancer. BMC Cancer. 2019 Dec;19(1):491.

58. Hauffe L, Picard D, Musa J, Remke M, Grünewald TGP, Rotblat B, et al. Eukaryotic translation initiation factor 4E binding protein 1 (EIF4EBP1) expression in glioblastoma is driven by ETS1- and MYBL2-dependent transcriptional activation. Cell Death Discov. 2022 Feb 28;8(1):91.

59. Chen Y, Lu A, Hu Z, Li J, Lu J. ERBB3 targeting: A promising approach to overcoming cancer therapeutic resistance. Cancer Lett. 2024 Sep;599:217146.

60. Zhao J, Zeng X, Hou SX. Glutamate-ammonia ligase promotes lung cancer cell growth through an enzyme-independent upregulation of CaMK2G under a glutamine-sufficient condition [Internet]. 2019 [cited 2025 Feb 8]. Available from: http://biorxiv.org/lookup/doi/10.1101/818575

61. Jiang Q, Li Y, Cai S, Shi X, Yang Y, Xing Z, et al. GLUL stabilizes N-Cadherin by antagonizing β-Catenin to inhibit the progresses of gastric cancer. Acta Pharm Sin B. 2024 Feb;14(2):698–711.

62. Cluntun AA, Lukey MJ, Cerione RA, Locasale JW. Glutamine Metabolism in Cancer: Understanding the Heterogeneity. Trends Cancer. 2017 Mar;3(3):169–80.

63. Shen L, Jiang S, Yang Y, Yang H, Fang Y, Tang M, et al. Pan-cancer and single-cell analysis reveal the prognostic value and immune response of NQO1. Front Cell Dev Biol. 2023 Jul 31;11:1174535.

64. Oh ET, Park HJ. Implications of NQO1 in cancer therapy. BMB Rep. 2015 Nov;48(11):609–17.

65. Janacova L, Stenckova M, Lapcik P, Hrachovinova S, Bouchalova P, Potesil D, et al. Catechol-O-methyl transferase suppresses cell invasion and interplays with MET signaling in estrogen dependent breast cancer. Sci Rep. 2023 Jan 23;13(1):1285.

66. Hashimoto Y, Shiina M, Maekawa S, Kato T, Shahryari V, Kulkarni P, et al. Suppressor effect of catechol-O-methyltransferase gene in prostate cancer. Culig Z, editor. PLOS ONE. 2021 Sep 29;16(9):e0253877.

67. Zhang Y, Ma X, Liu C, Bie Z, Liu G, Liu P, et al. Identification of HSPD1 as a novel invasive biomarker associated with mitophagy in pituitary adenomas. Transl Oncol. 2024 Mar;41:101886.

68. Kang BH, Shu CW, Chao JK, Lee CH, Fu TY, Liou HH, et al. HSPD1 repressed E-cadherin expression to promote cell invasion and migration for poor prognosis in oral squamous cell carcinoma. Sci Rep. 2019 Jun 20;9(1):8932.

69. Yu JS, Chen YT, Chiang WF, Hsiao YC, Chu LJ, See LC, et al. Saliva protein biomarkers to detect oral squamous cell carcinoma in a high-risk population in Taiwan. Proc Natl Acad Sci. 2016 Oct 11;113(41):11549–54.

70. Li X shan, Xu Q, Fu X yang, Luo W sheng. Heat Shock Protein 60 Overexpression Is Associated with the Progression and Prognosis in Gastric Cancer. Cappello F, editor. PLoS ONE. 2014 Sep 10;9(9):e107507.

71. Cappello F, David S, Rappa F, Bucchieri F, Marasà L, Bartolotta TE, et al. The expression of HSP60 and HSP10 in large bowel carcinomas with lymph node metastase. BMC Cancer. 2005 Dec;5(1):139.

72. Zhou C, Sun H, Zheng C, Gao J, Fu Q, Hu N, et al. Oncogenic HSP60 regulates mitochondrial oxidative phosphorylation to support Erk1/2 activation during pancreatic cancer cell growth. Cell Death Dis. 2018 Feb 7;9(2):161.

73. Zhang J, Zhou X, Chang H, Huang X, Guo X, Du X, et al. Hsp60 exerts a tumor suppressor function by inducing cell differentiation and inhibiting invasion in hepatocellular carcinoma. Oncotarget. 2016 Oct 12;7(42):68976–89.

74. Dlamini Z, Marima R, Hull R, Syrigos KN, Lolas G, Mphahlele L, et al. Genomics and molecular analysis of RPL9 and LIAS in lung cancer: Emerging implications in carcinogenesis. Inform Med Unlocked. 2021;25:100698.

75. Baik IH, Jo GH, Seo D, Ko MJ, Cho CH, Lee MG, et al. Knockdown of RPL9 expression inhibits colorectal carcinoma growth via the inactivation of Id-1/NF-κB signaling axis. Int J Oncol. 2016 Nov;49(5):1953–62.

76. Fu W, Lin Y, Bai M, Yao J, Huang C, Gao L, et al. Beyond ribosomal function: RPS6 deficiency suppresses cholangiocarcinoma cell growth by disrupting alternative splicing. Acta Pharm Sin B. 2024 Sep;14(9):3931–48.

77. Yang X, Xu L, Yang Y e, Xiong C, Yu J, Wang Y, et al. Knockdown of ribosomal protein S6 suppresses proliferation, migration, and invasion in epithelial ovarian cancer. J Ovarian Res. 2020 Dec;13(1):100.

78. Khalaileh A, Dreazen A, Khatib A, Apel R, Swisa A, Kidess-Bassir N, et al. Phosphorylation of Ribosomal Protein S6 Attenuates DNA Damage and Tumor Suppression during Development of Pancreatic Cancer. Cancer Res. 2013 Mar 15;73(6):1811–20.

79. Gambardella V, Gimeno-Valiente F, Tarazona N, Ciarpaglini CM, Roda D, Fleitas T, et al. NRF2 through RPS6 Activation Is Related to Anti-HER2 Drug Resistance in *HER2* - Amplified Gastric Cancer. Clin Cancer Res. 2019 Mar 1;25(5):1639–49.

80. Shirakawa Y, Hide T, Yamaoka M, Ito Y, Ito N, Ohta K, et al. Ribosomal protein S6 promotes stem-like characters in glioma cells. Cancer Sci. 2020 Jun;111(6):2041–51.

81. El Khoury W, Nasr Z. Deregulation of ribosomal proteins in human cancers. Biosci Rep. 2021 Dec 22;41(12):BSR20211577.

82. NCBI. INSIG1 insulin induced gene 1 [Homo sapiens (human)] - Gene [Internet]. National Institutes of Health (NIH). Available from: https://www.ncbi.nlm.nih.gov/gene/3638

83. Jiang W, Liu P, Li X. G9A performs important roles in the progression of breast cancer through upregulating its targets. Oncol Lett. 2017 Jun;13(6):4127–32.

84. Rye MB, Bertilsson H, Andersen MK, Rise K, Bathen TF, Drabløs F, et al. Cholesterol synthesis pathway genes in prostate cancer are transcriptionally downregulated when tissue confounding is minimized. BMC Cancer. 2018 Dec;18(1):478.

85. Akiyama K, Ohga N, Maishi N, Hida Y, Kitayama K, Kawamoto T, et al. The F-prostaglandin receptor is a novel marker for tumor endothelial cells in renal cell carcinoma. Pathol Int. 2013 Jan;63(1):37–44.

86. NCBI. NDRG1 N-myc downstream regulated 1 [Homo sapiens (human)]- Gene [Internet]. National Institutes of Health (NIH). Available from: https://www.ncbi.nlm.nih.gov/gene/10397

87. Park KC, Paluncic J, Kovacevic Z, Richardson DR. Pharmacological targeting and the diverse functions of the metastasis suppressor, NDRG1, in cancer. Free Radic Biol Med. 2020 Sep;157:154–75.

88. Joshi V, Lakhani SR, McCart Reed AE. NDRG1 in Cancer: A Suppressor, Promoter, or Both? Cancers. 2022 Nov 22;14(23):5739.

89. Kotepui K, Kotepui M, Majima HJ, Tangpong J. Association between NDRG1 protein expression and aggressive features of breast cancer: a systematic review and meta-analysis. BMC Cancer. 2023 Oct 19;23(1):1003.

90. Wu YN, Su X, Wang XQ, Liu NN, Xu ZW. The roles of phospholipase C-β related signals in the proliferation, metastasis and angiogenesis of malignant tumors, and the corresponding protective measures. Front Oncol. 2023 Jul 28;13:1231875.

91. Bertagnolo V, Benedusi M, Brugnoli F, Lanuti P, Marchisio M, Querzoli P, et al. Phospholipase C-β2 promotes mitosis and migration of human breast cancer-derived cells. Carcinogenesis. 2007 Aug;28(8):1638–45.

92. Wang S, Xie D, Yue H, Li G, Jiang B, Gao Y, et al. Phospholipase C Beta 2 as a Key Regulator of Tumor Progression and Epithelial-Mesenchymal Transition via PI3K/AKT Signaling in Renal Cell Carcinoma. Biomedicines. 2025 Jan 26;13(2):304.

93. Wang Y, Wang J, Gao J, Ding M, Li H. The expression of SERPINE1 in colon cancer and its regulatory network and prognostic value. BMC Gastroenterol. 2023 Feb 8;23(1):33.

94. Ju Y, Wang Z, Wang Q, Jin S, Sun P, Wei Y, et al. Pan-cancer analysis of SERPINE1 with a concentration on immune therapeutic and prognostic in gastric cancer. J Cell Mol Med. 2024 Aug;28(15):e18579.

95. Jevrić M, Matić IZ, Krivokuća A, Đorđić Crnogorac M, Besu I, Damjanović A, et al. Association of uPA and PAI-1 tumor levels and 4G/5G variants of PAI-1 gene with disease outcome in luminal HER2-negative node-negative breast cancer patients treated with adjuvant endocrine therapy. BMC Cancer. 2019 Dec;19(1):71.

96. Nakatsuka E, Sawada K, Nakamura K, Yoshimura A, Kinose Y, Kodama M, et al. Plasminogen activator inhibitor-1 is an independent prognostic factor of ovarian cancer and IMD-4482, a novel plasminogen activator inhibitor-1 inhibitor, inhibits ovarian cancer peritoneal dissemination. Oncotarget. 2017 Oct 27;8(52):89887–902.

97. Becker M, Szarvas T, Wittschier M, Vom Dorp F, Tötsch M, Schmid KW, et al. Prognostic impact of plasminogen activator inhibitor type 1 expression in bladder cancer. Cancer. 2010 Oct;116(19):4502–12.

98. Zubac DP, Wentzel-Larsen T, Seidal T, Bostad L. Type 1 plasminogen activator inhibitor (PAI-1) in clear cell renal cell carcinoma (CCRCC) and its impact on angiogenesis, progression and patient survival after radical nephrectomy. BMC Urol. 2010 Dec 3;10:20.

99. Sotiropoulos G, Kotopouli M, Karampela I, Christodoulatos GS, Antonakos G, Marinou I, et al. Circulating plasminogen activator inhibitor-1 activity: a biomarker for resectable non-small cell lung cancer? J BUON Off J Balk Union Oncol. 2019;24(3):943–54.

100. Weatherly LM, Shane HL, Lukomska E, Baur R, Anderson SE. Systemic toxicity induced by topical application of perfluoroheptanoic acid (PFHpA), perfluorohexanoic acid (PFHxA), and perfluoropentanoic acid (PFPeA) in a murine model. Food Chem Toxicol Int J Publ Br Ind Biol Res Assoc. 2023 Jan;171:113515.

101. Placencio VR, DeClerck YA. Plasminogen Activator Inhibitor-1 in Cancer: Rationale and Insight for Future Therapeutic Testing. Cancer Res. 2015 Aug 1;75(15):2969–74.

102. Gopal S, Veracini L, Grall D, Butori C, Schaub S, Audebert S, et al. Fibronectin-guided migration of carcinoma collectives. Nat Commun. 2017 Jan 19;8(1):14105.

103. Nam JM, Onodera Y, Bissell MJ, Park CC. Breast Cancer Cells in Three-dimensional Culture Display an Enhanced Radioresponse after Coordinate Targeting of Integrin α5β1 and Fibronectin. Cancer Res. 2010 Jul 1;70(13):5238–48.

104. Shi K, Wang S lin, Shen B, Yu F qiang, Weng D feng, Lin J hua. Clinicopathological and prognostic values of fibronectin and integrin αvβ3 expression in primary osteosarcoma. World J Surg Oncol. 2019 Dec;17(1):23.

105. Wang JP, Hielscher A. Fibronectin: How Its Aberrant Expression in Tumors May Improve Therapeutic Targeting. J Cancer. 2017;8(4):674–82.

106. Yi W, Xiao E, Ding R, Luo P, Yang Y. High expression of fibronectin is associated with poor prognosis, cell proliferation and malignancy via the NF-κB/p53-apoptosis signaling pathway in colorectal cancer. Oncol Rep. 2016 Nov;36(6):3145–53.

107. Efthymiou G, Saint A, Ruff M, Rekad Z, Ciais D, Van Obberghen-Schilling E. Shaping Up the Tumor Microenvironment With Cellular Fibronectin. Front Oncol. 2020 Apr 30;10:641.

108. Tan X, Gong W, Chen B, Gong B, Hua Z, Zhang S, et al. Downregulation of fibronectin 1 attenuates ATRA-induced inhibition of cell migration and invasion in neuroblastoma cells. Mol Cell Biochem. 2021 Oct;476(10):3601–12.

109. Zhan S, Li J, Wang T, Ge W. Quantitative Proteomics Analysis of Sporadic Medullary Thyroid Cancer Reveals FN1 as a Potential Novel Candidate Prognostic Biomarker. The Oncologist. 2018 Dec 1;23(12):1415–25.

110. Tang NH, Chen YL, Wang XQ, Li XJ, Wu Y, Zou QL, et al. N-terminal and C-terminal heparin-binding domain polypeptides derived from fibronectin reduce adhesion and invasion of liver cancer cells. BMC Cancer. 2010 Dec;10(1):552.

111. Chang YC, Chiou J, Yang YF, Su CY, Lin YF, Yang CN, et al. Therapeutic Targeting of Aldolase A Interactions Inhibits Lung Cancer Metastasis and Prolongs Survival. Cancer Res. 2019 Sep 15;79(18):4754–66.

112. Song J, Li H, Liu Y, Li X, Shi Q, Lei Q, et al. Aldolase A Accelerates Cancer Progression by Modulating mRNA Translation and Protein Biosynthesis via Noncanonical Mechanisms. Adv Sci. 2023 Sep;10(26):2302425.

113. Ji S, Zhang B, Liu J, Qin Y, Liang C, Shi S, et al. ALDOA functions as an oncogene in the highly metastatic pancreatic cancer. Cancer Lett. 2016 Apr 28;374(1):127–35.

114. Jiang Z, Wang X, Li J, Yang H, Lin X. Aldolase A as a prognostic factor and mediator of progression via inducing epithelial-mesenchymal transition in gastric cancer. J Cell Mol Med. 2018 Sep;22(9):4377–86.

115. Ambrosio MR, Piccaluga PP, Ponzoni M, Rocca BJ, Malagnino V, Onorati M, et al. The alteration of lipid metabolism in Burkitt lymphoma identifies a novel marker: adipophilin. PloS One. 2012;7(8):e44315.

116. Fujimoto M, Matsuzaki I, Yamamoto Y, Yoshizawa A, Warigaya K, Iwahashi Y, et al. Adipophilin expression in cutaneous malignant melanoma. J Cutan Pathol. 2017 Mar;44(3):228–36.

117. Matsubara J, Honda K, Ono M, Sekine S, Tanaka Y, Kobayashi M, et al. Identification of adipophilin as a potential plasma biomarker for colorectal cancer using label-free quantitative mass spectrometry and protein microarray. Cancer Epidemiol Biomark Prev Publ Am Assoc Cancer Res Cosponsored Am Soc Prev Oncol. 2011 Oct;20(10):2195–203.

118. Ostler DA, Prieto VG, Reed JA, Deavers MT, Lazar AJ, Ivan D. Adipophilin expression in sebaceous tumors and other cutaneous lesions with clear cell histology: an immunohistochemical study of 117 cases. Mod Pathol Off J U S Can Acad Pathol Inc.

2010 Apr;23(4):567–73.

119. Liu W, Liu X, Liu Y, Ling T, Chen D, Otkur W, et al. PLIN2 promotes HCC cells proliferation by inhibiting the degradation of HIF1α. Exp Cell Res. 2022 Sep;418(1):113244.

120. TRIB3 Gene - Tribbles Pseudokinase 3 [Internet]. GeneCards. Available from: https://www.genecards.org/cgi-bin/carddisp.pl?gene=TRIB3

121. Xing Y, Luo P, Hu R, Wang D, Zhou G, Jiang J. TRIB3 Promotes Lung Adenocarcinoma Progression via an Enhanced Warburg Effect. Cancer Manag Res. 2020;12:13195–206.

122. Yu J jiao, Zhou D dan, Yang X xiao, Cui B, Tan F wei, Wang J, et al. TRIB3-EGFR interaction promotes lung cancer progression and defines a therapeutic target. Nat Commun. 2020 Jul 21;11(1):3660.

123. Yin Z, Wu D, Shi J, Wei X, Jin N, Lu X, et al. Identification of ALDH3A2 as a novel prognostic biomarker in gastric adenocarcinoma using integrated bioinformatics analysis. BMC Cancer. 2020 Nov 4;20(1):1062.

124. Lv Q, Shi J, Miao D, Tan D, Zhao C, Xiong Z, et al. miR-1182-mediated ALDH3A2 inhibition affects lipid metabolism and progression in ccRCC by activating the PI3K-AKT pathway. Transl Oncol. 2024 Feb;40:101835.

125. Dong H, He L, Sun Q, Zhan J, Li J, Xiong X, et al. Inhibit ALDH3A2 reduce ovarian cancer cells survival via elevating ferroptosis sensitivity. Gene. 2023 Aug;876:147515.

126. ALDH3A2 Gene - Aldehyde Dehydrogenase 3 Family Member [Internet]. GeneCards. Available from: https://www.genecards.org/cgi-bin/carddisp.pl?gene=ALDH3A2

127. Ayroldi E, Cannarile L, Delfino DV, Riccardi C. A dual role for glucocorticoid-induced leucine zipper in glucocorticoid function: tumor growth promotion or suppression? Cell Death Dis. 2018 Apr 26;9(5):463.
